# Integrating lipid metabolism, pheromone production and perception by Fruitless and Hepatocyte Nuclear Factor 4

**DOI:** 10.1126/sciadv.adf6254

**Published:** 2023-06-30

**Authors:** Jie Sun, Wen-Kan Liu, Calder Ellsworth, Qian Sun, Yufeng Pan, Yi-Chun Huang, Wu-Min Deng

**Affiliations:** ^1^ Department of Biochemistry and Molecular Biology, Tulane University School of Medicine, New Orleans, LA 70112, USA.; ^2^ Tulane Cancer Center, Tulane University School of Medicine, New Orleans, LA 70112, USA.; ^3^ Department of Entomology, Louisiana State University, Baton Rouge, LA 70803, USA.; ^4^ The Key Laboratory of Developmental Genes and Human Disease, School of Life Science and Technology, Southeast University, Nanjing 210096, China.

## Abstract

Sexual attraction and perception are crucial for mating and reproductive success. In *Drosophila melanogaster*, the male-specific isoform of Fruitless (Fru), Fru^M^, is a known master neuro-regulator of innate courtship behavior to control the perception of sex pheromones in sensory neurons. Here, we show that the non–sex-specific Fru isoform (Fru^COM^) is necessary for pheromone biosynthesis in hepatocyte-like oenocytes for sexual attraction. Loss of Fru^COM^ in oenocytes resulted in adults with reduced levels of cuticular hydrocarbons (CHCs), including sex pheromones, and show altered sexual attraction and reduced cuticular hydrophobicity. We further identify *Hepatocyte nuclear factor 4* (*Hnf4*) as a key target of Fru^COM^ in directing fatty acid conversion to hydrocarbons. *Fru* or *Hnf4* depletion in oenocytes disrupts lipid homeostasis, resulting in a sex-dimorphic CHC profile that differs from *doublesex-* and *transformer-*dependent CHC dimorphism. Thus, Fru couples pheromone perception and production in separate organs to regulate chemosensory communications and ensure efficient mating behavior.

## Teaser

Fruitless and lipid metabolism regulator HNF4 integrate pheromone production and perception to ensure robust courtship behavior.

## INTRODUCTION

Chemical sensing, regarded from an evolutionary perspective as the oldest one, is common to all organisms, which are surrounded by a world full of odors emitted from conspecific or heterospecific individuals and the environment (*1*). Chemical communication is fundamental to social behaviors such as conspecific recognition, courtship, aggression, aggregation, and avoidance. Many animals, including insects, rely on chemical cues to locate and select appropriate mating partners for reproductive success. Insect chemical communication involves the emission and perception of chemical cues (pheromones) and requires the coordination of different organs that are involved in pheromone biosynthesis and sensing, respectively (*2*). *Drosophila*, with its long tradition in behavioral studies, is an ideal model to explore the evolution and diversity of pheromones associated with the diverse array of social behaviors, and the ethological context connecting chemical communication to behavioral and systemic processes. Although substantial progress has been made in the *Drosophila* model during the past decades in unraveling the receptors and neural circuits for pheromone detection that give rise to innate behaviors, whether and how pheromone emission and perception are regulated in a coordinated manner remain to be explored.

Insect cuticular hydrocarbons (CHCs), derived from long-chain fatty acids (LCFAs), are important for desiccation resistance (*3*–*5*) and starvation resistance (*6*). Some CHCs function as sex pheromones for mate recognition (*7*–*11*). As in other insects, specialized hepatocyte-like oenocytes, located in the inner surface of the abdominal cuticle, are the primary site for the biosynthesis of very LCFAs (VLCFAs) and VLCFA-derived hydrocarbons in *Drosophila melanogaster* (*10*, *12*–*14*). Oenocytes are also the major site for reactive oxygen species metabolism, proteasome-mediated protein catabolism, xenobiotic metabolism, ketogenesis, and peroxisomal β-oxidation (*15*–*21*). Although the biosynthetic pathway for CHCs is active in both male and female oenocytes, the CHC profile shows sexual dimorphism (*22*). In most populations of *D. melanogaster*, sexually dimorphic expression of enzymes such as *elongase F* (*eloF*) and *desaturase F* (*desatF*) leads to female-biased enrichment of several CHCs including 7,11-HD and 7,11-ND (*23*, *24*). Further proof that the CHC composition could be affected by sex-determination genes has been shown by ectopic expression of *transformer* (*tra*) in male oenocytes, which up-regulates *eloF* and feminizes the CHC profile (*24*, *25*). However, the upstream molecular regulation mechanism of how these enzymes are associated with the sexual dimorphism of CHC biosynthesis remains elusive.

Pheromone perception in *Drosophila* requires the neuronal function of *fruitless* (*fru*), a master regulator of the sexually dimorphic neural circuits that underlie sexual dimorphic patterns of courtship (*26*–*29*), aggression (*30*–*32*), sleep (*33*), and other behaviors. The *fru* gene spans more than 150 kb of the genome and harbors P1 to P4 promoters (*34*, *35*). The distal P1 promoter is dedicated to the expression of male-specific Fru proteins (Fru^M^), whereas P2 to P4 promoters are involved in the production of non–sex-specific Fru proteins (Fru^COM^) (fig. S1) (*35*–*37*). Compared with the well-characterized involvement of Fru^M^ in neuronal behavior, the precise function of Fru^COM^ in development and behavior is less clear (*38*, *39*).

In this study, we report that Fru^COM^ is required for CHC biosynthesis in adult oenocytes of both sexes for chemical communication and desiccation resistance. Silencing of *fru* in oenocytes resulted in desiccation-sensitive adults with accompanying social behavioral changes. We further identify evolutionarily conserved hepatocyte nuclear factor 4 (HNF4) as a key target of Fru^COM^ to inhibit steatosis and to direct fatty acid conversion to hydrocarbons in oenocytes. Thus, *fru* is involved in both pheromone biosynthesis and perception by distinct splicing isoforms in separate organs, bringing sexual attraction and perception under the control of a single gene.

## RESULTS

### *Fru* is required in both the nervous system and oenocytes for innate courtship behavior

**Fig. 1.**
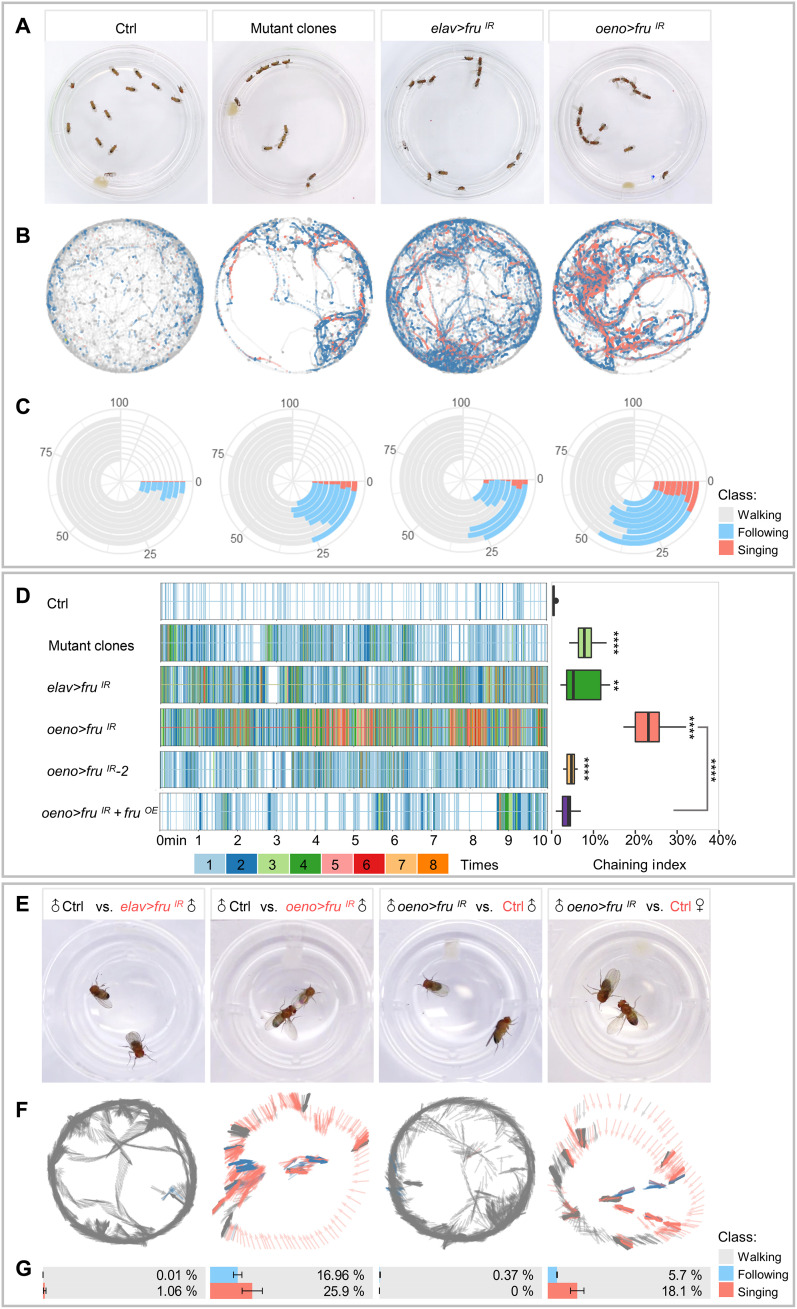
Loss of *fru* function in the nervous system or oenocytes alters male courtship behavior. (**A**) Representative movie screenshots of fly groups with indicated genotypes showing male-male following and chaining behaviors in experimental groups. Thirteen male adults of the same genotype were collected and placed in one chamber. (**B**) Event maps generated from representative movies of indicated genotypes (1.5-min movie per map). (**C**) Polarized bar plots illustrating the ratio of each detected behavior. Each bar plot was generated from eight independent biological replicates (10-min movie per replicate). (**D**) Rug plots showing the chaining events detected from 10-min movies. The *x* axis refers to the time of the movie. Each vertical line with different color stands for the number of chaining events detected in a single frame. The corresponding color to the number of events is illustrated in the legend. The box plots of the chaining index are quantitative statistics of eight independent biological replicates. (**E**) Representative movie screenshots of pair-housed flies with indicated genotypes (black: active party; red: passive beheaded party; control: *oeno-Gal4/+*). The trajectory and behavioral classification of the active party are shown in (**F**). The arrow indicates the head orientation of the active party flies. The color of the arrow indicates different behavior statuses (gray: resting/walking; blue: following; salmon: singing) (1.5-min movie per map). Bar plots with error bars are the quantitative statistics of 10-min movies of eight independent biological replicates in (**G**). Data are represented as means ± SEM. *P* values are calculated using one-way analysis of variance (ANOVA) (D) and two-tailed unpaired *t* test (G) followed by Holm-Sidak multiple comparisons. ns, not significant; *P < 0.01 and ****P < 0.0001.

To understand the function of Fru^COM^ isoforms, we generated mosaic flies with whole-body clonal mutation of *fru* using a recently developed mosaic analysis by guide (g)RNA-induced crossing-over (MAGIC) technique, which generates mosaic animals containing genetically distinct populations of cells, based on DNA double-strand breaks produced by CRISPR-Cas9 (*40*). The double gRNA design allowed us to eliminate all Fru isoforms in mutant clones randomly induced by a heat-shock inducible Cas9 (fig. S1). Male flies bearing *fru* MAGIC clones, which were induced during the early third larval instar, exhibited male-male courtship and male chaining behavior (Fig. 1, A to D, and movie S1). This behavior is similar to Fru^M^ mutant flies reported previously (*41*, *42*). The male-male courtship phenotype was reproduced when we used the Gal4/UAS system (*43*) to knock down *fru* expression with a pan-neuronal driver *elav-Gal4* and a double-stranded (ds) RNA targeting exon C3, which is present in all Fru isoforms (Fig. 1, A to D, fig. S1, and movie S2).

To better assay the composite behavior phenotype of different *fru* loss-of-function (LOF) flies, we developed a machine learning–based automatic fly-behavioral detection and annotation (MAFDA) system to track flies and identify multiple classes of behavior such as chasing, singing, and copulating (fig. S2, A to E). The MAFDA platform facilitates the visualization of complex behavioral phenotypes under a temporal and spatial overlay, and quantitative comparison of behavioral indexes among different genetic backgrounds. When compared with idtracker.ai, a widely used animal trajectory tracking platform (*44*, *45*), MAFDA ran and processed the same 10-min video (*n* = 13 flies) markedly faster (6 hours on idtracker versus 20 min on MAFDA). The MAFDA platform also has the function of real-time shooting and tracking recognition. In addition, the trajectories identified by idtracker tended to have long straight lines, while the same trajectories identified using MAFDA were continuous and smooth, even when the fly objects changed directions (fig. S2, F and H). By checking back at the video, we found that most of the long straight lines were caused by object loss or ID switch during tracking of fast-moving and overlapping objects and were much less frequent in MAFDA than idtracker (fig. S2, J and I).

Using the MAFDA platform, we found that the male-male following, singing, and chaining behaviors exhibited in flies with MAGIC-induced *fru* mutant clones were comparable to or more pronounced than neuronal *fru* knockdown (Fig. 1D and fig. S3C). The severity of the behavior defects displayed by MAGIC *fru* mosaic flies is puzzling, as chances of random mutant clones hitting specific neurons are relatively small. We wondered whether the regulation of courtship behavior by Fru is not limited to the nervous system, as biosynthesis and release of pheromones in peripheral tissues play an important role as well. We therefore decided to test whether loss of *fru* may also interfere with pheromone production and release.

Oenocytes, which perform certain lipid processing functions, are the principal site for the synthesis of VLCFA-derived CHCs, including sex pheromones, that are associated with a variety of social behaviors in *D. melanogaster* (*10*, *12*, *13*). Using two reported oenocyte-Gal4 drivers (*promE-Gal4* and *OK72-Gal4*) (*46*, *47*) to knock down *fru* expression, respectively, we found that male adult flies displayed extensive male-male following and chaining behaviors (hereafter, we refer *promE-Gal4* as *oeno-Gal4*; Fig. 1, A to D, fig. S3C, and movie S3). MAFDA analysis revealed that the male-male courtship behavior exhibited by *oeno > fru*^*IR*^ flies was highly pronounced (45% following, 25% chaining, and 8% singing; Fig. 1D and fig. S3C) and could be alleviated by Fru^COM^ overexpression (*UAS-fru^COMB^*) in oenocytes (Fig. 1D and fig. S3, C and E), indicating that the defect was caused by *fru* silencing. To avoid possible off-target effects of the dsRNA, additional independent *fru* RNA interference (RNAi) lines were used and showed similar male-male courtship phenotypes (Fig. 1D and fig. S3F). As revealed by MAFDA analysis, the degree of male-male courtship behavior by distinct RNAi lines was consistent with their respective knockdown efficiency (Fig. 1D and figs. S3C and S6D). In addition, we applied the gene switch (GS), a modified Gal4/UAS system that allows temporal and spatial control of transgene expression by adding the drug RU486 to food (*48*, *49*). RU486 was added to induce *fru* knockdown after pupal eclosion to avoid a possible role of *fru* on oenocyte development from its larval progenitors. The behavioral phenotypes were similar between *fru* knockdown flies with or without GS in oenocytes (fig. S3G). Together, these findings suggest that *fru* is also required in oenocytes to maintain male-male repulsion.

To verify that the male-male courtship behavior in *oeno > fru^IR^* flies was caused by changes in sexual signaling, such as pheromone production, rather than pheromone perception, we performed the single-pair mating experiment by placing a male fly with a headless target in the same chamber. The wild-type male was attracted to the headless male with *fru* depletion in oenocytes (*oeno > fru^IR^*), but not the headless male lacking Fru in pan-neurons (*elav > fru^IR^*) (Fig. 1, E to G). By contrast, the male fly with oenocyte-specific *fru* knockdown (*oeno > fru^IR^*) selected the decapitated wild-type female to initiate courtship but averted the wild-type male (Fig. 1, E to G). These results indicate that, while males lacking Fru in oenocytes maintain the ability in mate choice, they are attractive to wild-type males. Furthermore, to determine whether females with oenocyte depletion of Fru (*oeno > fru^IR^*) also show altered sexual attraction, we performed the two-choice courtship assay by putting a wild-type male and two headless female targets (*oeno > fru^IR^* and control *oeno-Gal4/+*) in the same chamber. MAFDA analysis revealed that the courtship index (CI) for *oeno > fru^IR^* females was ~60% of that of the control group (fig. S3, H and I), suggesting that females with oenocyte *fru* knockdown show reduced sexual attractiveness to male flies. Together, these results demonstrate that a deficiency of *fru* in oenocytes modifies sexual attraction in both sexes.

### Flies with *fru* depletion in oenocytes exhibit aberrant social behavior

**Fig. 2.**
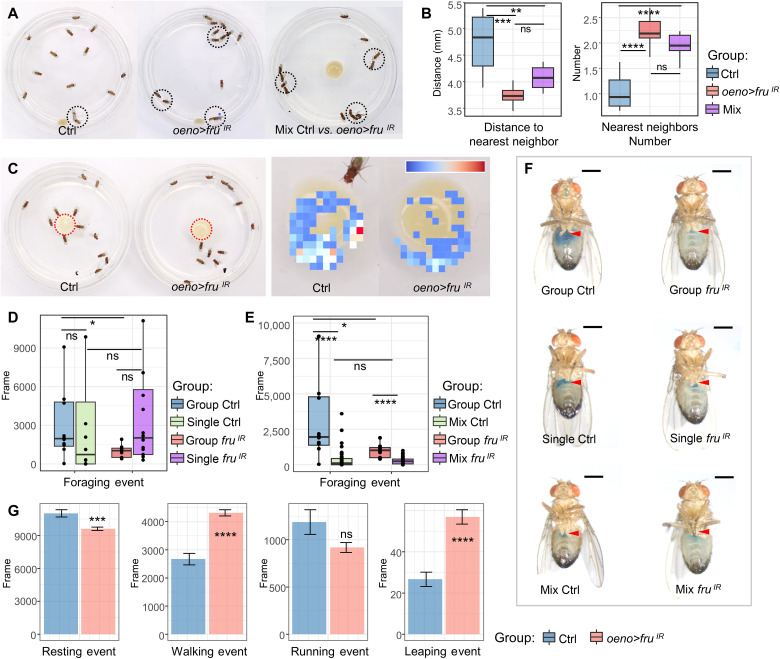
Social behavior changes in male flies with *fru* depletion in oenocytes. (**A**) Male social space behavior analysis. Representative screenshots of an open-field assay indicate that flies in experimental groups tend to stay close together (black circles). (**B**) Quantification of the distance of each fly to its nearest neighbor and the number of surrounding neighbors of each fly for both control (*oeno-Gal4/+*) and *oeno > fru^IR^* males (*n* = 8 groups of 13 male flies for each genotype) (10-min movie per replicate). (**C**) Control (*oeno-Gal4/+*) and *oeno > fru^IR^* males were analyzed for the total number of social interactions in a “competition-for-food” assay. The heatmap on the right shows the degree to which flies gather in the food area. (**D**) Quantification of the total number of feeding times of the flies for control (*oeno-Gal4/+*) and *oeno > fru^IR^* males under group and single housing conditions (*n* = 10 groups of 13 flies for each group, *n* = 16 single-housing fly) (1-hour video per replicate). (**E**) Quantification of the total number of feeding times of the flies for control (*oeno-Gal4/+*) and *oeno > fru^IR^* males under group and mix housing conditions (*n* = 10 groups of 13 flies for each group, *n* = 8 mix-housing flies) (1-hour video per replicate). (**F**) Measurement of solid food intake in adults using a dye tracer (2 hours). Scale bars, 500 μm. (**G**) Locomotion activity of control (*oeno-Gal4/+*) and *oeno > fru^IR^* males was monitored. From left to right, bar plots show resting events, walking events, running events, and jumping events of flies, respectively (*n* = 8 independent experiments with 13 flies per genotype). For all paradigms, data are represented as means ± SEM. *P* values are calculated using one-way ANOVA (B), Wilcoxon rank sum test (D) and (E), and two-tailed unpaired *t* test (G) followed by Holm-Sidak multiple comparisons. Asterisks illustrate statistically significant differences between conditions. *P < 0.05, **P < 0.01, ***P < 0.001, and ****P < 0.0001.

In *Drosophila*, innate social behaviors driven by pheromones include aggregation, male courtship, and female postmating behaviors (*50*). To test whether *fru* knockdown affects social aggregation, we measured the distance between individual male flies and their nearest neighbors: their “social space” (*51*), within a social group. *Oeno > fru^IR^* flies, on average, showed reduced social space and had more nearby neighbors when compared with the control (Fig. 2, A and B), suggesting that *fru* expression in oenocytes is necessary for flies to maintain their social distance. When we mixed male flies with two genotypes (six males of each genotype), the controls (*oeno-Gal4/+*) were attracted to *oeno > fru^IR^* flies, consistent with the paired experiment (Fig. 1, E and F), leading to a decrease in the overall social distance within the mixed population (Fig. 2, A and B). In addition, *oeno > fru^IR^* male flies showed reduced foraging behavior in a fixed time window (1 hour) (Fig. 2C). MAFDA analysis revealed that the foraging index of the *oeno > fru^IR^* males was ~60% of the control group, while there was no notable difference in the foraging behavior between the single-housing flies of these two genotypes (Fig. 2D). When the two genotypes were mixed in the same chamber, the foraging behavior of both genotypes decreased markedly (Fig. 2E). Measurement of solid food intake in adults via a dye tracer further revealed that flies with *fru* depletion ingested less food than wild-type flies over the same time period (2 hours). The food-intake difference was greater in the group setting than the single-house setting, suggesting that *fru* is required in oenocytes for social foraging behavior (Fig. 2F). To rule out that the observed social behavior changes are caused by impaired motor ability, we further examined their locomotion activity during the daytime. Compared with the control group, *oeno > fru^IR^* flies were more active, showing a 10% reduction in resting, a 35% increase in walking, and a 55% increase in leaping by MAFDA analysis (Fig. 2G), suggesting that the observed social behavior changes are not caused by impaired motor ability. Together, these data provide evidence that *fru* is required in oenocytes for innate social behavior.

### Fru function is necessary for CHC production

**Fig. 3.**
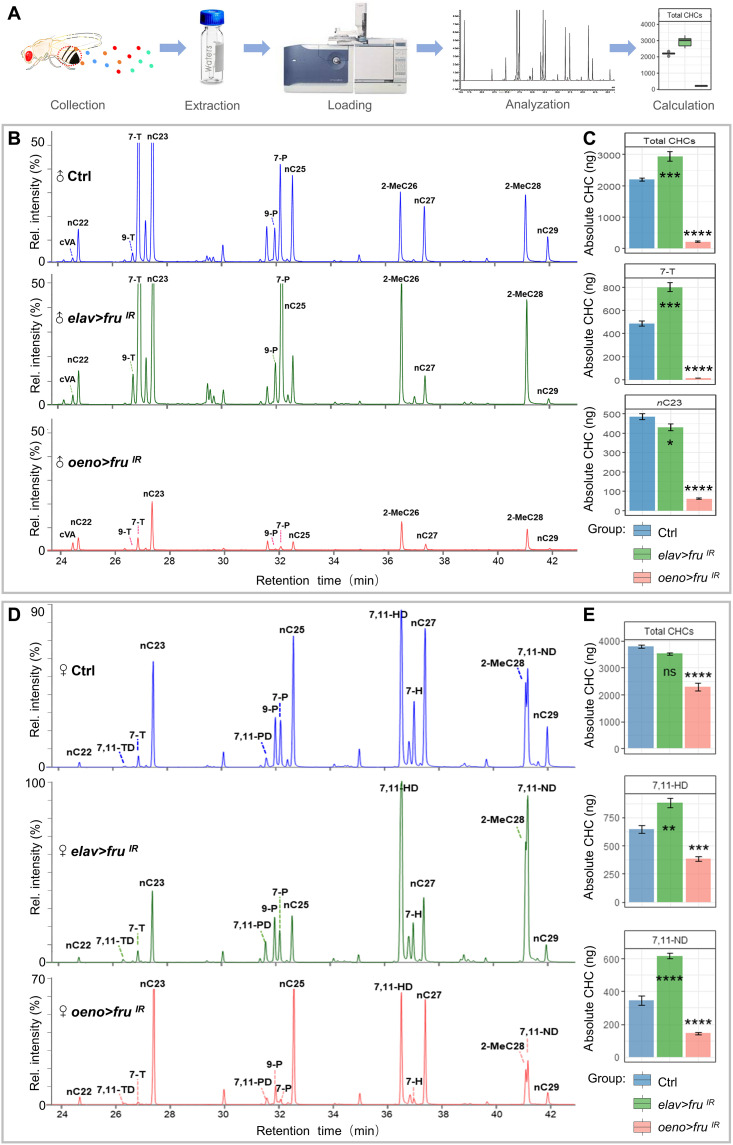
Fru is required in the oenocytes for the biosynthesis of cuticular hydrocarbons. (**A**) Schematic of cuticular hydrocarbon extraction and GC-MS analysis. (**B**) CHCs from adult male flies of each genotype were analyzed using GC-MS. Compared with the control (*oeno-Gal4/+*) and *elav > fru^IR^* males, *oeno > fru^IR^* males exhibit noticeably lower levels of CHCs. (**C**) The absolute contents of total hydrocarbons, male key pheromones (7-T and nC23) carried by a single male were calculated by the loading of internal standards. (**D**) CHCs from females of each genotype were analyzed using GC-MS. Compared to *controls* and *elav > fru^IR^* females, *oeno > fru^IR^* females exhibit lower levels of CHCs. (**E**) The absolute contents of total hydrocarbons, female key pheromones (7,11-HD and 7,11-ND) carried by a single female were calculated by the loading of internal standards. Data are represented as means ± SEM. *P* values are calculated using one-way ANOVA followed by Holm-Sidak multiple comparisons. Asterisks illustrate statistically significant differences between conditions. *P < 0.05, **P < 0.01, ***P < 0.001, and ****P < 0.0001.

In *Drosophila*, CHCs act primarily as pheromones and play fundamental roles in sexual attraction or repulsion (*10*, *52*). Using gas chromatography–mass spectrometry (GC-MS) analysis, we identified 46 distinct chromatogram peaks from the cuticle extracts of wild-type adults and determined the chemical identities of their corresponding hydrocarbons (Fig. 3A and fig. S4, A and B). Subsequent analysis of CHC profiles by GC-MS and comparison of the main cuticular extracts of *elav > fru^IR^* and *oeno > fru^IR^* males revealed that the levels of total hydrocarbons were reduced by 90% when *fru* was knocked down in oenocytes, while *fru*-loss in the nervous system showed a CHC profile largely resembled the wild-type control (Fig. 3B and fig. S5A). Comparison of the CHC content in *oeno > fru^IR^* male flies revealed that 7-tricosene (7-T), the principal nonvolatile male pheromone (*10*), was reduced by 97% (Fig. 3C). Because 7-T mediates repulsion to other males and prevent male-male interactions (*46*, *53*), the drastic reduction of 7-T explains why wild-type males show a strong preference of males with *fru* knockdown in the oenocyte (Fig. 1, A to D). In addition, *oeno > fru^IR^* males showed a 87% decrease of *n*-tricosane (*n*C23), which has been shown to negatively regulate courtship and mating in sexually mature *Drosophila suzukii* (*54*), though its behavioral implication in *D. melanogaster* is not clear.

CHCs are highly sexually dimorphic in *D. melanogaster*, with many of the compounds present in one sex but absent in the other, while shared compounds often differ between sexes (*55*). To determine whether *fru* knockdown also alters the CHC profile in female flies, we performed the GS-MS analysis and found that the total CHCs were reduced by 40% in *oeno > fru^IR^* females when compared with the control (Fig. 3E). Different from the male flies, the changes in the CHC profile fall mainly into the CHCs with the chain length beyond C26, which include the female pheromones 7,11-HD and 7,11-ND (Fig. 3D) (*46*). These results explain why wild-type females are more sexually attractive than females with *fru* knockdown in oenocytes (fig. S3I).

The hydrophobic properties of CHCs protect insects from transpirational water loss through insect epicuticles and play a major role in desiccation resistance (*13*, *56*). In *oeno > fru^IR^* flies, we found a substantial reduction of multiple long-chain *n*-alkanes, including *n*-heptacosane (*n*C27) and *n*-nonacosane (*n*C29) (Fig. 3, B to E, and fig. S5A). To test the hydrophobicity of these flies, both male and female adults were dehydrated and incubated in water to assess defects in the hydrophobic coating of the cuticle. While control carcasses remained at the surface of the liquid following a 4-hour incubation, *oeno > fru^IR^* carcasses sank below the surface (fig. S5, B and C). Together, these results suggest that *fru* is required in both male and female oenocytes for CHC and sex pheromone production.

### Fru^COM^, not Fru^M^, is expressed in the oenocytes

*fru* is a complex locus with sophisticated precise spatiotemporal control of transcription through four distinct promoters (fig. S1) (*34*, *35*, *39*). Translation of P1 transcripts in males produces the male-specific Fru^M^ proteins that have an amino-terminal extension of 101 amino acids preceding the BTB domain, whereas transcripts from the P2 to P4 promoters encode a set of non–sex-specific Fru^COM^ proteins that have essential functions in the development of both sexes (fig. S1) (*36*). All Fru proteins are putative transcription factors containing a common BTB N-terminal domain and five alternatively spliced C terminus varying in the number and sequence of Zn-finger DNA binding domains (A, B, C, D, or E) (fig. S1) (*34*, *35*, *57*).

To determine which Fru isoforms are expressed in oenocytes, we generated a polyclonal antibody recognizing an epitope in exon C3, which is present in both Fru^COM^ and Fru^M^ isoforms (fig. S1). Similar to the two reported Fru^COM^ antibodies (*39*, *58*), this new anti-Fru^COM^ antibody labeled a large number of cells in the central nervous system (CNS) of mature third instar larvae at the peak of Fru expression in both sexes (fig. S6A), and the signal was noticeably reduced when *fru* was knocked down in the CNS with two independent RNAi lines (BDSC#31593 and VDRC330035; fig. S6, A and B). Staining adult oenocytes with the new anti-Fru^COM^ showed a strong signal in the nuclei of both male and female flies, whereas the Fru^M^-specific antibody (*59*) detected no signal (fig. S6, C and D). Fru^COM^ staining was lost in oenocytes with *fru-*RNAi knockdown or with MAGIC-induced *fru* depletion (fig. S6, D and E). Analysis of anti-Fru^COM^ signals in oenocytes showed that the two *fru-*RNAi lines induced different knockdown efficiencies, which were confirmed by reverse transcription quantitative polymerase chain reaction (RT-qPCR) analysis with *actin5c-Gal4* for whole-body *fru* knockdown (fig. S6G). The difference in knockdown efficiency is probably responsible for the difference in the severity of the male-male courtship behavior phenotypes (Fig. 1, A to D, and fig. S6F). Together, these results suggest that Fru^COM^, not Fru^M^, is expressed in oenocytes in both sexes.

### Fru^COM^ is required for proper expression of genes involved in hydrocarbon biosynthesis in oenocytes

**Fig. 4.**
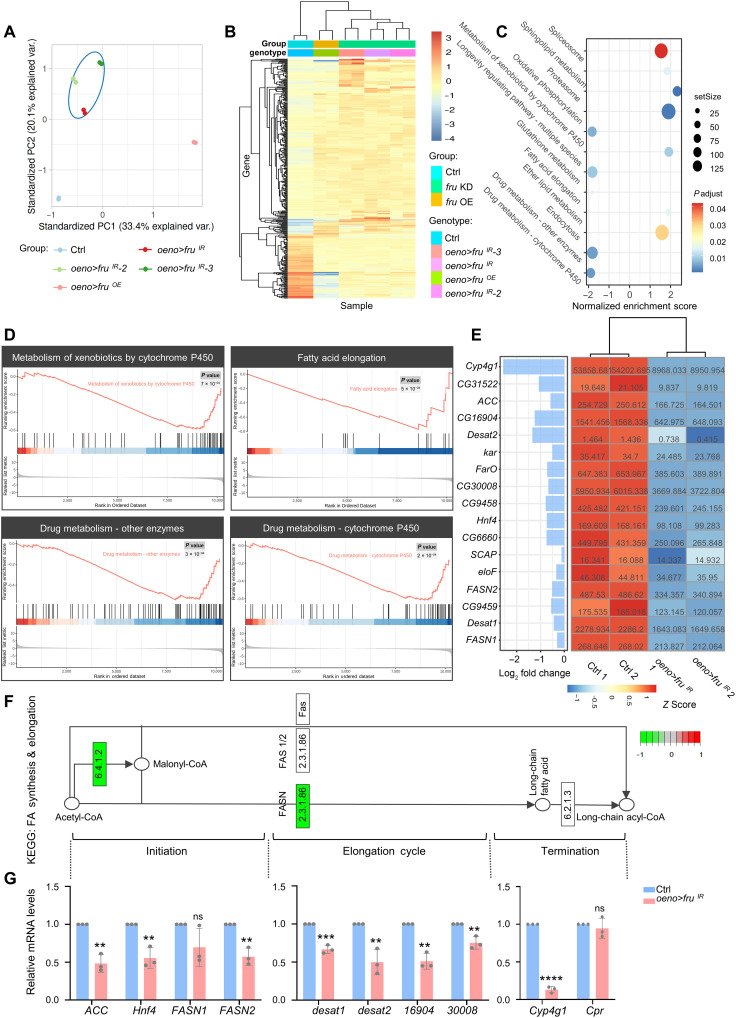
Bulk RNA-seq analysis to identify genes affected by *fru* knockdown in oenocytes. (**A**) PCA plot shows that the biological replicates of each genotype are highly consistent. The datasets from *fru* knockdown induced by three independent RNAi lines show good clustering. (**B**) Heatmap of expression of differentially expressed genes identified from bulk RNA-seq. Gene expression is shown in normalized log_2_ counts per million. Differentially expressed genes were selected on the basis of the threshold of *P* ≤ 0.001 and ≥4-fold change. (**C**) Kyoto Encyclopedia of Genes and Genomes (KEGG) pathway enrichment analysis. Pathway enrichment of differentially expressed genes. The *y* axis indicates the pathway name, and the *x* axis indicates the fold change. (**D**) Gene set enrichment analysis for interpreting gene expression data of four metabolic pathways. (**E**) Down-regulation of VLCFA/hydrocarbon biosynthesis genes in oenocytes with *fru* knockdown from two independent replicates. Colors in the heatmap correspond to the scaled FPKM which is shown as a number in each cell. (**F**) The FA synthesis & elongation pathway from KEGG, with green boxes indicating genes down-regulated in this subset, and RT-qPCR analysis of genes in the VLCFA/hydrocarbon metabolic pathway (controls: blue bars; *oeno > fru^IR^*: salmon bars) (**G**). Transcript levels are normalized to *Rp49* mRNA and presented relative to the level of controls. Asterisks illustrate statistically significant differences between conditions. **P < 0.01, ***P < 0.001, and ****P < 0.0001.

LCFA biosynthesis is predominantly derived from palmitate, which is synthesized by *acetyl-CoA carboxylase* (*ACC*) and *fatty acid synthase 2* (*FASN2*) (*60*). After that, they are elongated by the successive action of elongases, desaturases, and decarbonylate enzymes, before being converted to hydrocarbons (*13*, *56*, *61*) (fig. S7). The enzymes involved in the hydrocarbon biosynthetic pathway are highly conserved in insects (*22*). To determine how Fru^COM^ affects hydrocarbon biosynthesis, we generated multiple RNA sequencing (RNA-seq) datasets from adult male oenocytes with *fru* knockdown by three independent *fru*-RNAi lines with corresponding control samples and a Fru^COM^ overexpression line, which were then subjected to principal components analysis (PCA). On the PCA plot, the three independent *fru*-RNAi datasets clustered together and separated from the control and Fru^COM^ overexpression samples (Fig. 4A). Hierarchical cluster analysis of significantly different genes (*P* ≤ 0.001 and ≥4-fold change) revealed that the control, *fru* knockdown, and Fru^COM^ overexpression groups formed distinct clusters with clear differences at the whole transcriptome level (Fig. 4B). Kyoto Encyclopedia of Genes and Genomes (KEGG) pathway enrichment analysis revealed that the majority of down-regulated genes in the *fru* knockdown group were related to metabolic regulation, including fatty acid elongation, xenobiotic, and drug metabolism (Fig. 4C). These genes and pathways show conserved functions in *Drosophila* oenocytes and the mammalian liver (*62*). Using clusterProfiler, we performed gene set enrichment analysis (GSEA) (*63*) and identified subtle but coordinated expression changes of metabolic pathways following *fru* knockdown, revealing down-regulation of genes in xenobiotic metabolism, drug metabolic, and fatty acid elongation pathways (Fig. 4D and fig. S7). Notably, genes involved in various steps of VLCFA and hydrocarbon biosynthesis, including *ACC*, *Hnf4*, *FASN2*, *desaturase1* (*desat1*), *cytochrome P450 4 g1* (*Cyp4g1*), and multiple *elongases* (*CG16904* and *CG30008*), all showed substantial down-regulation in the *fru* knockdown group (Fig. 4E). Decreased transcription of these genes was confirmed by RT-qPCR analysis of RNAs extracted from male oenocytes (Fig. 4, F and G). These findings suggest that Fru is required for the developmental induction of key genes in the VLCFA/hydrocarbon biosynthetic pathway in adulthood.

### Fru^COM^ controls *Hnf4* expression in oenocytes to maintain lipid homeostasis

**Fig. 5.**
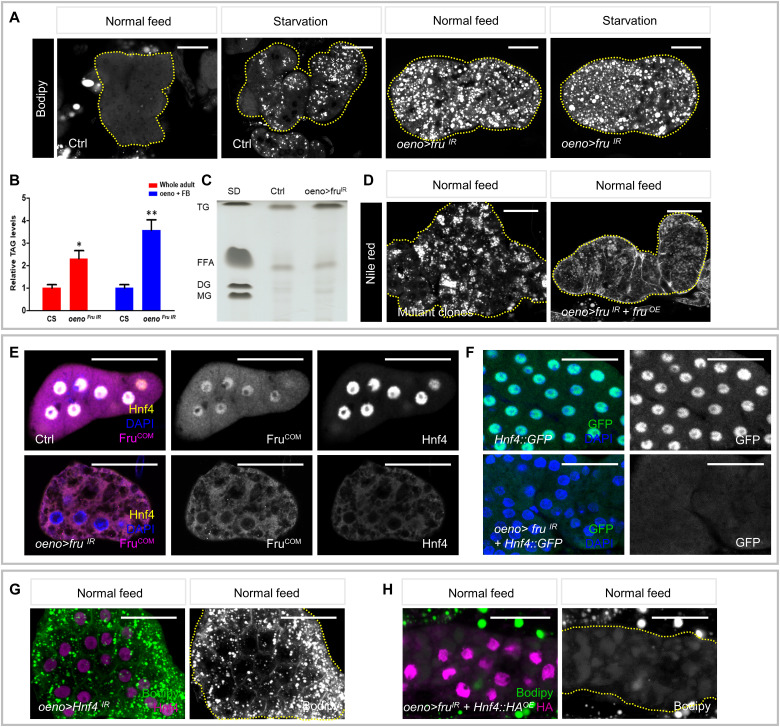
Fru controls HNF4 expression in the oenocytes to inhibit steatosis. (**A**) Bodipy stains are depicted for oenocytes dissected from control and *oeno > fru^IR^* males raised on standard diet (SD) and starved for 7 days after emergence. Oenocytes are outlined with a yellow dotted line. (**B**) Triglyceride levels were measured in 7-day-old control and *oeno > fru^IR^* reared on a SD after emergence. Metabolite levels are normalized to total protein and presented relative to the amount in control animals. Red indicates that a decapitated whole body was used as the sample material, and blue indicates that only adipose tissue [fat body (FB) and oenocytes] was used. CS, control system. (**C**) Thin layer chromatography analysis shows TAG and free fatty acid (FFA) levels in adipose tissue of control and *oeno > fru^IR^* males. DG, diacylglycerol. MG, Monoacylglycerol. (**D**) Nile red stains are depicted for oenocytes dissected from *fru* mutant clones and *oeno > fru^IR^ + fru^OE^* males cultured on SD. (**E**) *fru* knockdown in oenocytes resulted in decreased HNF4 protein levels. Top: Fru (magenta) colocalized with HNF4 (yellow) in control oenocytes. Bottom: The level of HNF4 was noticeably reduced in oenocytes of *fru* knockdown. (**F**) Green fluorescent protein (GFP)–tagged HNF4 from the endogenous *Hnf4* promoter is highly expressed in oenocytes, but the GFP signal is lost in oenocytes with *fru* knockdown. (**G**) Bodipy (green) and anti-HNF4 (magenta) stains are depicted for oenocytes dissected from *oeno > Hnf4^IR^* males raised on SD. (**H**) Bodipy stains are depicted for oenocytes of *oeno > fru*^*I*R*+*^*Hnf4^OE^* males cultured on SD. Scale bars, 10 μm. DAPI, 4′,6-diamidino-2-phenylindole. *P < 0.05 and **P < 0.01.

The hepatocyte-like oenocytes accumulate lipid droplets (LDs) as a normal response to fasting and this readout of steatosis also indicates abnormal lipid metabolism in fed flies (*16*, *46*). We noticed LD accumulation in oenocytes in both starved and fed *oeno > fru^IR^* flies, suggesting that *fru* is required for preventing steatosis independent of nutritional status (Fig. 5A). To determine whether *fru* knockdown interferes with systemic lipid homeostasis, we measured the triacylglycerol (TAG) levels in *oeno > fru^IR^* male adults and found that the whole-body TAG level of these flies was 2.4-fold of the control (Fig. 5B). In addition, thin layer chromatography (TLC) of adipose tissue revealed that the TAG content, but not free fatty acids, was noticeably increased when *fru* was depleted in oenocytes (Fig. 5C). Consistently, flies bearing *fru* MAGIC clones exhibited similar LDs accumulation, and *fru* depletion–induced steatosis could be alleviated by Fru^COM^ overexpression in oenocytes (Fig. 5D). These results indicate that Fru functions in oenocytes to prevent steatosis and to maintain systemic lipid homeostasis.

Among the down-regulated genes induced by *fru* depletion, we focused on *Hnf4*, which has been reported to be necessary for maintaining lipid homeostasis and promoting fatty acid conversion to VLCFA and hydrophobic hydrocarbons in oenocytes (*64*). To determine whether HNF4 acts downstream of Fru, we first used an anti-HNF4 antibody (*65*) and found that the HNF4 protein level was obviously down-regulated in *fru*-silenced oenocytes (Fig. 5E and fig. S8A). Consistently, a HNF4 protein trap line with green fluorescent protein (GFP)–tagged endogenous HNF4 (*66*) showed loss of the GFP signal in oenocytes with *fru* depletion (Fig. 5F), further suggesting that HNF4 expression is regulated by *fru* in oenocytes. To determine whether *Hnf4* is functionally downstream of *fru* in oenocytes, the dsRNAs of *Hnf4* (*Hnf4^IR^*) were targeted to oenocytes by *oeno-Gal4*. As expected, the knockdown of *Hnf4* in oenocytes resulted in similar steatosis, as reported previously (*64*), whereas HNF4 overexpression alleviated lipid accumulation induced by *fru* depletion (Fig. 5, G and H). Together, these results suggest that Fru^COM^ regulates HNF4 expression in oenocytes to inhibit steatosis and maintain systemic lipid homeostasis.

### *Hnf4* is essential in mediating *fru*-regulated CHC biosynthesis and courtship behavior

**Fig. 6.**
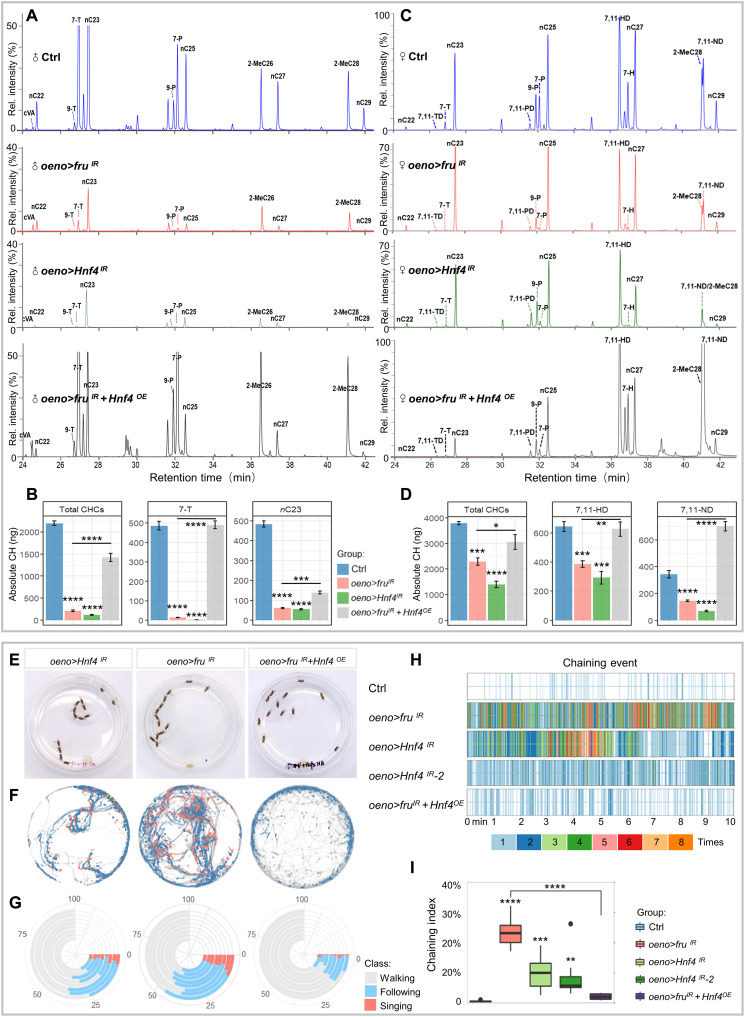
Fru controls HNF4 expression in the oenocytes to maintain CHC biosynthesis and innate courtship behavior. (**A**) GC-MS analysis of CHCs from male adults in control (*oeno-Gal4/+*), *oeno > fru^IR^*, *oeno > Hnf4^IR^*, and *oeno > fru^IR^ + Hnf4^OE^*. (**B**) The absolute contents of total hydrocarbons, male key pheromones (7-T and nC23) carried by a single male were calculated by the loading of internal standards. (**C**) GC-MS analysis of CHCs from female adults of controls (*oeno-Gal4/+*), *oeno > fru^IR^*, *oeno > Hnf4^IR^*, and *oeno > fru^IR^ + Hnf4^OE^* females. (**D**) The absolute contents of total hydrocarbons, female key pheromones (7,11-HD and 7,11-ND) carried by a single female were calculated by the loading of internal standards. Data are represented as means ± SEM. *P* values are calculated using one-way ANOVA followed by Holm-Sidak multiple comparisons. Asterisks illustrate statistically significant differences between conditions. *P < 0.05, **P < 0.01, ***P < 0.001, and ****P < 0.0001. (**E**) Representative movie screenshots of fly groups with indicated genotypes. Thirteen male adults of the same genotype were collected and placed in one chamber. (**F**) Event maps generated from representative movies of indicated genotypes (1.5-min movie per map). (**G**) Polarized bar plots illustrating the ratio of each detected behavior. Each bar plot was produced from eight independent biological replicates (10-min movie per replicate). (**H**) Rug plots showing the chaining events detected from 10-min movies. The *x* axis refers to the time of the movie. Each vertical line with different colors indicates the number of chaining events detected in a single frame. The corresponding color to the number of events is illustrated in the legend. (**I**) The box plots of the chaining index are quantitative statistics of eight independent biological replicates.

To determine whether *fru*-regulated CHC production is mediated by HNF4, we used GC-MS to measure hydrocarbon levels in flies with HNF4 depletion in oenocytes (*oeno > Hnf4^IR^*). Expectedly, these flies also show substantial decreases in total CHCs in both sexes (a 94% decrease in males and a 62% decrease in females), similar to flies with *fru* knockdown in oenocytes (Fig. 6, A to D). Notably, the knockdown of *Hnf4* also resulted in a defective synthesis of sex pheromones in both sexes, including male pheromone 7-T (99% decrease) and *n*C23 (89% decrease) as well as female pheromone 7,11-HD (66% decrease) and 7,11-ND (80% decrease) (Fig. 6, A to D). In addition, *oeno > Hnf4^IR^* flies also showed a marked reduction of multiple long-chain *n*-alkanes (Fig. 6, A to D), resulting in defects in the hydrophobic coating of cuticles in both male and female adults (fig. S8B). Next, we asked whether reintroducing HNF4 in oenocytes with *fru* knockdown would restore the CHC profile and found that the total CHC levels were restored to 71 and 79% of the control males and females, respectively. It is noteworthy that the major sex pheromones in both male and female flies, especially 7-T and 7,11-HD, were well recovered and comparable to the levels of the control flies (Fig. 6, A to D).

The impairment of CHC biosynthesis suggests that flies with *Hnf4* knockdown in oenocytes may also have changed courtship behavior patterns. To test this, we applied the MAFDA platform to analyze the courtship behavior of male flies with oenocyte *Hnf4* knockdown using two independent *Hnf4-RNAi* lines. As expected, these flies exhibited pervasive male-male courtship and chaining behaviors, similar to *fru*-depleted males (Fig. 6, E to I; fig. S8, C and D; and movie S4). Misexpression of HNF4 in oenocytes with *fru* knockdown alleviated the male-male courtship and male-chaining phenotypes when compared with *fru* knockdown alone (Fig. 6, E to I, fig. S8D, and movie S5), highly consistent with their respective CHC profiles (Fig. 6A). Together, these results suggest HNF4 is a key factor downstream of Fru in oenocytes to control pheromone and CHC production and its down-regulation is accountable for the behavior phenotypes displayed by *oeno > fru^IR^* flies.

### *dsx* and *fru* depletion induce two distinct sex-dimorphic CHC profiles

**Fig. 7.**
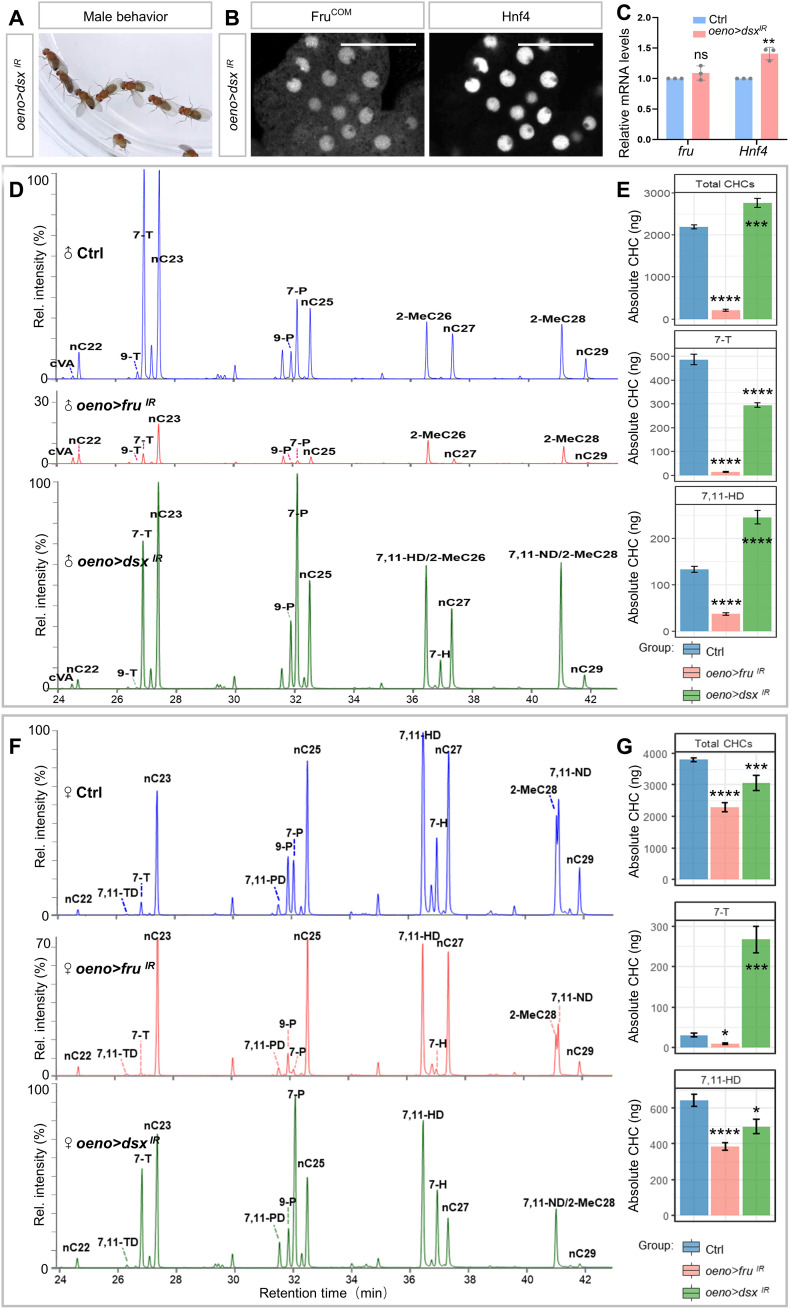
Fru does not act downstream of Dsx in regulating CHC biosynthesis. (**A**) Silencing of *dsx* in oenocytes causes male chaining behavior. (**B**) Immunostaining shows that Fru^COM^ and HNF4 protein levels remain high in oenocytes with *dsx* knockdown. Scale bar, 10 μm. (**C**) Transcript levels of *fru* and *Hnf4* were analyzed by RT-qPCR in *dsx* knockdown oenocytes (control: blue bars; *oeno > dsx^IR^*: salmon bars). Transcript levels were normalized to Rp49 mRNA and presented relative to control levels. (**D**) CHCs from males of each genotype were analyzed using GC-MS. *oeno > fru^IR^* males exhibit noticeably lower levels of CHCs in the full spectrum than the control (*oeno-Gal4/+*). (**E**) The CHCs of *oeno > dsx^IR^* males, in contrast, exhibit a mixture of low levels of characteristic male hydrocarbons (7-T) and high levels of diene hydrocarbons (7,11-HD and 7,11-ND, characteristic of females). (**F**) GC-MS analysis of CHCs in female adults shows that oenocyte-specific knockdown (*oeno > fru^IR^*) resulted in lower levels of CHCs in the full spectrum than control females. (**G**) Knockdown of *dsx* in female oenocytes (*oeno > dsx^IR^*) resulted in lower female pheromones (diene hydrocarbons 7,11-HD and 7,11-ND) but high levels of male pheromone (7-T). Data are represented as means ± SEM. *P* values are calculated using two-tailed unpaired *t* test (C) and one-way ANOVA (E) and (G) followed by Holm-Sidak multiple comparisons. Asterisks illustrate statistically significant differences between conditions. *P < 0.05, **P < 0.01, ***P < 0.001, and ****P < 0.0001.

Although *fru* or *Hnf4* knockdowns in oenocytes induced substantial decreases of total CHCs in both male and female flies, the changes appeared sexually dimorphic. While males showed almost complete loss of CHCs, the reduction of CHCs in females was mostly on those with a chain length beyond C26 (Fig. 3, B to E). In insects, the sexual dimorphism of CHCs could be regulated by sex-determination genes, including *tra* and *dsx* (*23*, *67*–*69*). Consistently, knockdown of *dsx* in oenocytes caused male-chaining behavior (Fig. 7A). To test whether *fru* acts downstream of *dsx* in oenocytes, we first performed immunohistochemistry analyses using anti-HNF4 and anti-Fru^COM^ antibodies and found that the protein levels of HNF4 and Fru^COM^ were not noticeably changed in oenocytes with *dsx* knockdown (Fig. 7B). RT-qPCR analysis further showed that *dsx* knockdown did not cause obvious changes in *fru* transcripts but up-regulated *Hnf4* by 41% (Fig. 7C). Further CHC profiling revealed that oenocyte-specific depletion of *dsx* led to a 26% increase in total CHCs in males but a 19% decrease in females (Fig. 7, D to G). Consistent with these findings, *oeno > dsx^IR^* male flies showed better water repellency than controls, whereas females had impaired hydrophobic coating of the cuticle (fig. S9, A and B). And at the pheromone level, *dsx*-loss in oenocytes caused feminization of the pheromone profile in male flies and masculinization of the pheromone profile in females (Fig. 7, D to G). These findings reveal that, despite the phenotypic similarity in courtship behavior between *dsx*- and *fru*-deleted flies, the underlying mechanism of these two genes in regulating CHC and pheromone profiles is different (Fig. 7, D to G). The mechanism underlying the sex differences of CHC profile in *fru*- and *Hnf4*-depleted flies is yet to be determined. Nonetheless, our results suggest that *fru* is not a downstream target of *dsx* in regulating CHC biosynthesis in oenocytes.

## DISCUSSION

**Fig. 8.**
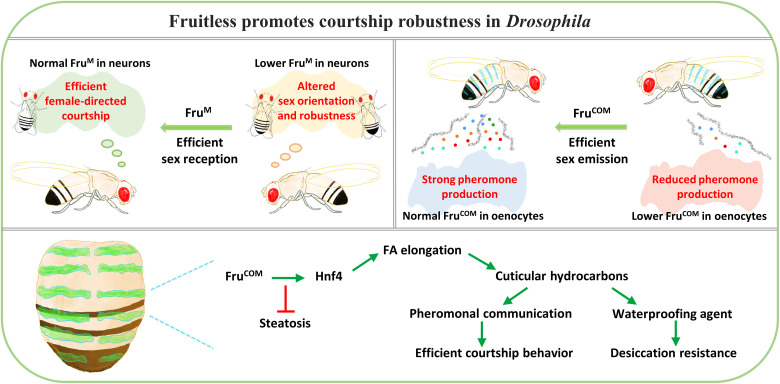
The model of *fruitless* regulates pheromone biosynthesis and perception. A schematic drawing to show that Fru regulates both pheromone production and perception through different isoforms (the male-specific Fru^M^ and the non–sex-specific Fru^COM^) expressed in different organs, thereby promoting the robustness and efficiency of courtship behavior. Fru^COM^ expression in oenocytes regulates HNF4 protein levels for the biosynthesis of sex pheromones, along with other cuticle hydrocarbons required for desiccation resistance.

This study reveals a combined control of pheromone production and perception by a zinc-finger transcription factor gene, *fru*, which regulates lipid homeostasis through an evolutionarily conserved gene *Hnf4*, thereby promoting the robustness and efficiency of courtship behavior in *D. melanogaster*. Such an integrated regulation of sexual attractiveness and sexual perception/execution by a single gene in distinct cells may have a reproductive advantage, as the recruitment of *fru* in certain insect species (*70*) could enhance both the emission and perception of sex-related cues simultaneously, which has stronger selective advantage than separate evolvement of each process (Fig. 8). Previously, *desat1* has been shown to be involved in both pheromone production and perception (*71*). A recent study found that the *Gr8a* gene, which belongs to the gustatory receptor gene family, is expressed specifically in male oenocytes and plays a role in the synthesis of certain male CHCs by regulating the level of Desat1 (*72*). On the basis of our RNA-seq analysis, *desat1* is a likely downstream target of *fru* in the oenocyte. It will be interesting to find out if *desat1* also acts downstream of *fru* in pheromone production and/or perception.

The *fru* gene locus contains a complex transcription unit with multiple promoters and alternative splicing isoforms (Fig. 4A). *fru* P1 transcripts have only been detected in the nervous system, their sex-specific protein product Fru^M^ is expressed in ∼2000 neurons to masculinize their structure and function (*73*). In contrast, Fru^COM^ is expressed in both neural and nonneural tissues. Its expression has been detected in neuroblasts in both male and female larval CNS (*39*). Fru^COM^ appears to be also required in the adult female brain to regulate female rejection behavior (*74*). Outside the nervous system, Fru^COM^ is detected in specific cell types in the reproductive system (*58*, *73*) and is necessary for the maintenance of germline stem cells and cyst stem cells in male gonads (*38*). Our study shows that, in pheromone-producing oenocytes, Fru^COM^, rather than Fru^M^, is expressed in both sexes and plays a key role in CHC biosynthesis (fig. S6, C and D). Although how the sex- and tissue-dynamic expression of Fru^M^ and Fru^COM^ is orchestrated remains to be resolved, the utilization of different transcriptional/splicing products of the same gene in pheromone production and perception may provide a potential advantage in coordinating these two related biological processes during development.

In insects, pheromone synthesis has coevolved with chemosensory perception. Numerous examples of correlated evolutionary changes between pheromone production and perception have been reported, with evolutionary pros and cons of sex-specific pheromones mirroring their sensory responses (*75*, *76*). The genetic mechanisms that control pheromone perception are best understood in *Drosophila* with >150 chemoreceptor proteins identified (*10*). The odorant receptor neurons (ORNs) that express ORs are specialized to detect the most volatile chemicals, including low-volatility pheromones (*77*). In Or47b ORNs, *fru* has been shown to regulate sensory plasticity and act as a downstream genomic coincidence detector (*78*–*81*). Although it is unclear whether *Hnf4* is regulated by *fru* in the nervous system, the neuronal function of *Hnf4* has been reported during neural stem cell differentiation and in the aging brain for β-oxidation of fatty acids (*82*, *83*). Impaired lipid homeostasis has been shown to be associated with disrupted neuronal integrity in human neurodegenerative diseases (*84*). It will be interesting to find out whether lipid homeostasis regulated by *Hnf4* is an integral part of the *fru-*regulated neural network in pheromone detection.

Many insect CHCs and pheromones undergo rapid evolution and show sexual dimorphism. Genetic manipulations of sex-determination genes such as *sxl*, *dsx*, or *tra* in *D. melanogaster* have been shown to alter the CHC profile (*47*, *85*–*88*). Gain of function or LOF *dsx* or *tra* induces either masculinization or feminization of CHCs, with major changes at sex-specific pheromones (Fig. 7, D to G) (*47*, *85*). The sexually dimorphic CHC patterns regulated by *dsx* or *tra* appear to stem from their regulation of DesatF and EloF expression, respectively (*23*), which promotes female long-chain hydrocarbon biosynthesis (Fig. 7, D and E) (*24*, *25*). Our study revealed an unusual sex-dimorphic CHC profile resulting from *fru* or *Hnf4* depletion in oenocytes (Figs. 3, B to E, and 6, A to D), which differs substantially from *sxl-*, *dsx-* or *tra-*dependent sexual dimorphism of the CHC profile (Fig. 7, D to G) (*69*). In contrast, male flies with *fru* or *Hnf4* knockdown have substantially reduced pheromones, and their behavioral phenotype is consistent with a previous report that wild-type males exhibit a higher preference for males without CHCs (similar to the *oeno > fru^IR^* generated in this study) over wild-type females (*46*). The regulatory hierarchy between *dsx* and *fru* may be different between tissues. In the CNS, transcripts of both *fru* and *dsx* undergo sex-specific splicing regulated by upstream genes in the *Drosophila* sex-determination hierarchy (*89*, *90*). In male gonads, expression of Fru^COM^ is regulated by *dsx* and independent of *tra* (*38*). However, in oenoctyes, we found that neither protein nor transcript levels of *fru* were noticeably changed when *dsx* was knocked down. Note that the transcript level of *Hnf4* was slightly up-regulated in *oeno > dsx^IR^*, which may contribute to the synthesis of long-chain feminizing pheromones in males (Fig. 7, B and C). Although our studies did not reveal whether *Hnf4* is a transcription target of Fru or Dsx in the oenocyte, previous studies have shown the existence of direct binding sites for Fru/Dsx on the *Hnf4* locus (*91*, *92*).

The evolutionary variation of CHCs among different *Drosophila* species is necessary for the maintenance of reproductive isolation. A class of dienes that serve as sex pheromones exhibit particularly diverse patterns between species with sexually monomorphic or dimorphic CHCs. *Drosophila* species with sexually dimorphic CHCs, such as *D. melanogaster*, *Drosophila sechellia*, and *Drosophila erecta*, produce long-chain dienes in females specifically (*8*, *55*, *87*). Species with sexually monomorphic CHCs can be divided into two categories: the one that produces long-chain dienes in both sexes, such as *Drosophila serrata*, *Drosophila pseudoobscura*, and *Drosophila persimilis* (*93*–*95*), and the other that does not produce dienes, such as *Drosophila simulans*, *Drosophila mauritiana*, *Drosophila yakuba*, *Drosophila teissieri*, *Drosophila orena*, and *Drosophila santomea* (*23*, *96*–*98*). The acquisition of a binding site for Dsx at the *DesatF* regulatory region is suggested to be responsible for the evolutionary transition from monomorphism to dimorphism (*23*). In *D. melanogaster*, the induction of masculinized females by *dsx*-loss is caused by down-regulation of DesatF expression in oenocytes, which leads to decreased synthesis of dienes and increased monoenes (such as 7-T and 7-P; Fig. 7, F and G) (*24*, *25*). Consistently, the knockdown of *desatF* in oenocytes also results in a decrease in dienes and an increase in monoenes (*99*), as insect CHCs are synthesized from a common pathway that uses acetyl-CoA as the substrate for chain elongation, the changes in dienes would have an indirect effect on monoenes (*100*). Although the *dsx* locus is highly conserved, *desatF* and its expression evolve rapidly. In species with monomorphic CHCs that do not express DesatF, such as *D. simulans*, genetic ablation of *dsx* will probably not affect 7-T significantly since this species does not synthesize diene CHCs.

Fru and HNF4, revealed from our studies, may play a broad role in connecting multiple steps of VLCFC metabolism and CHC biosynthesis, including fatty acid elongation, desaturation, decarbonylation, etc. (Fig. 4, E to G). A crucial downstream gene of Fru in oenocytes is probably *Cyp4g1*, which encodes a functionally conserved P450 enzyme involved in the terminal oxidation decarbonylation of CHCs (*64*). Oenocyte-specific knockdown of *Cyp4g1* causes an almost complete loss of all CHCs (*56*), a phenotype similar to the knockdown of *fru*. Depletion of *fru* in oenocytes markedly reduced the transcript level of *Cyp4g1* (Fig. 4, E to G).

*fru* and *dsx* may contribute to the evolution of CHCs and interspecies reproductive isolation through different mechanisms. The highly conserved Dsx isoforms mainly generate a small number of sexually dimorphic CHCs. Different from *dsx*, *fru* knockdown affects a wide range of alkanes, monoenes, and dienes (Figs. 3, B to E, and 6, A to D). Thus, the evolution of *fru* in insects may generate diverse amounts of CHCs across species, contributing to reproductive isolation to some extent. Note that only Fru^COM^, but not Fru^M^, is expressed in some insect species, such as Hawaiian flies (*101*), suggesting that the P1 promoter–derived Fru^M^ may be evolved later in males to couple pheromone perception with pheromone production, a process that may further enhance mating efficiency.

## MATERIALS AND METHODS

### Fly strains and genetics

Flies were maintained at 25°C, 60% relative humidity, and a 12-hour light/dark cycle. Adults and larvae were reared on a standard cornmeal and yeast-based diet unless otherwise noted. The Gal4/UAS–driven RNAi crosses were cultured at 25°C until eclosion, and then incubated at 29°C for 7 days, and the same culture conditions were also used for the control group.

*promE-Gal4* and *promE-GS-Gal4* (*oeno-specific GAL4*) were obtained from H. Bai (*102*). To evaluate the influence of *fru* knockdown on the efficiency of the *promE-GS-Gal4* line, we examined the fluorescent intensity of GFP in the oenocyte of *promE-GS > UAS-mCD8-GFP* and found that *fru* knockdown reduced GFP fluorescent intensity by ~58% (fig. S3A).

*UAS-fru^MA^* and *UAS-fru^COMB^* were from M. Arbeitman. *elav-Gal4* (BDSC#458), *UAS-fru^RNAi^* (BDSC#31593), *UAS*-*GFP* (BDSC#5413), *OK72-Gal4* (BDSC#6486), *Hnf4::GFP.FLAG* (BDSC#38649), *UAS-Hnf4^RNAi^* (BDSC#29375), *UAS-Hnf4^RNAi^* (BDSC#64988), *UAS-dsx^RNAi^* (BDSC#55646), *Act5C-GAL4* (BDSC#4414), and *W1118* (BDSC#5905) were obtained from the Bloomington *Drosophila* Stock Center. *UAS-fru-gRNA* (VDRC#342548), *UAS-fru^RNAi^* (VDRC#330035), and *UAS-fru^RNAi^* (VDRC#105005) were obtained from the Vienna *Drosophila* Resource Center. *UAS-Hnf4::HA* (F000144) was obtained from FlyORF (Zurich ORFeome Project).

RU486 (mifepristone, Thermo Fisher Scientific) was dissolved in 95% ethanol, and then added to standard food at a final concentration of 100 μM for all the experiments. For activation of the GS Gal4 driver, flies were fed on RU486 food for seven consecutive days, unless otherwise noted.

To temporally induce *fru* MAGIC clones, the early third instar larvae of *w; UAS-fru-gRNA/Act5C-Gal4; HS-Cas9/+* were heat-shocked for 30 min at 37°C and examined on day 7 after eclosion. The *HS-Cas9* stock was provided by C. Han (*40*).

### Behavioral assays

For the single-pair courtship assay, a tester male and a target fly (both on day 7 after eclosion) were gently aspirated into a round two-layer chamber (diameter: 1 cm; height: 3 mm per layer) to allow the courtship test. The CI, the percentage of observed time a fly performed any courtship step, was used to measure courtship between the tester male and the target fly. Each test was performed for 1 hour.

For the male chaining assay, the tester males were loaded into large round chambers (diameter: 4 cm; height: 3 mm) by cold anesthesia. The chaining index, which is the percentage of observed time during which at least three flies engaged in courtship, was used to measure the courtship behavior in groups of 13 males.

For the two-choice courtship assay, a standard courtship assay was used to test male preference (i.e., female attractiveness) between *fru* knockdown (*oeno > fru^IR^*) and the control (*oeno-Gal4/+*) females. Assays were performed under both normal and dark conditions. For each measurement, two subject females for comparison were decapitated and placed on the opposite sides of a single well in a standard 12-well cell culture plate containing standard fly medium, and a 5- to 7-day-old *w1118* virgin male was subsequently aspirated into the cell. The video recording lasted for 1 hour to record the courtship behaviors (including orientation, wing vibration, and attempted copulation) of the male fly directed toward each female. The assays were conducted at 25°C at night. To control for individual variability, male preference was shown as the percentage of time the *w1118* male was courting the *fru* knockdown female divided by the total courtship time.

For the social space assay, the tester males were loaded into large round chambers (diameter: 4 cm; height: 3 mm) by cold anesthesia. Flies were allowed to acclimate for 10 min, and then, digital videos were collected after the flies reached a stable position (up to 20 min). Digital images were imported in YOLOv5 and an automated measure of the nearest neighbor to each fly was determined using our own MAFDA system.

For the foraging assay, the tester males were analyzed using horizontal circular chambers: for group-housing testing (diameter: 4 cm; height: 3 mm); for single-housing testing (diameter: 1 cm; height 3 mm). Flies were allowed to acclimate for 1 hour; digital video images were then collected after the flies reached a stable position (up to 20 min). Digital images were imported in YOLOv5, and the location of food was given manually, using our own MAFDA system to determine the number of foraging for each fly through its distribution and dwell time.

### Development of MAFDA system

The present study used YOLOv5 (https://ultralytics.com/yolov5), a state-of-the-art object detection algorithm, as the platform for training a dataset consisting of 800 well-annotated pictures. The dataset was subjected to augmentation techniques, including flipping, merging, rotating, brightening, and random-salt masking, which resulted in the generation of approximately 140,000 labeled flies, 130,000 labeled heads, and the collection of around 40,000 chasing events, 8700 wing-expansion events, and 1600 mountings. The training process was conducted on the LONI server using the default hyperparameters and the YOLOv5x.pt. file as the initial weight. The model was trained with a batch size of 80, using 500 epochs, and an image size of 640 × 640 pixels, running on Tesla V100 hardware for over a day. The trained model achieved exceptional accuracy in detecting and categorizing the various events of interest, as evidenced by the results obtained in this study. To identify chaining events, we used a criterion based on two or more independent chase events targeting the same object.

A tracking algorithm was developed for monitoring the movement of flies between adjacent frames, which is based on “distance-sort.” Each fly is identified and assigned a specific ID by the system in the first frame. In subsequent frames, the position of each fly is paired with its nearest neighbor in the previous frame, and the fly inherits its ID. If a target fly becomes lost, its ID remains associated with the position of the previous frame until the target reappears. In cases where an ID switch occurs, the switch is identified and corrected manually. To facilitate the correction of ID switches, we developed a graphical user interface (GUI) software based on Python 3.8 and Kivy 2.00. The software allows users to easily and accurately correct ID-switch errors, ensuring that the tracking algorithm remains robust and reliable.

We compared the tracking performance of the MAFDA platform with idtracker.ai (*45*). The installation of idtracker.ai was carried out in accordance with the instructions provided on the GitHub platform. The GUI was used to initiate tracking on four separate videos, each containing 13 flies, while the same four videos were also tracked using the MAFDA platform. Both idtracker and MAFDA platforms were run on the same computer equipped with a single NVIDIA GeForce RTX 2080 GPU. idtracker.ai required approximately 6 hours per video to complete tracking, in contrast to the MAFDA platform, which required only 20 min per video. Furthermore, idtracker.ai was observed to have more frequent ID switching during the tracking of fast-moving and overlapping objects.

To generate a real-time event map of fly behaviors in real-time using the MAFDA system, each fly in each frame of the video (captured at a rate of 30 frames per second) is marked as a dot, and the frames are subsequently overlaid. To distinguish between different behavioral events, we use a color-coding scheme where each event is represented by a unique colored dot. To avoid over-representation, we generated event maps using 1.5-min video segments, which effectively exhibit the trajectories and behavioral classifications of flies in a given group.

The behavior indexes (including the chasing index, singing index, and chaining index) were calculated with the detected behavior events divided by the maximum possible value for that particular event to occur. The formula for calculating the behavior index is as follows: Index = B(d)/B(max) × 100%, where B(d) is the number of detected behavior events and B(max) is the maximum possible value for that event to occur. Chasing and singing B(max) equate to the total number of fly number (N) in the group, and chaining B(max) equates to N−1. For example, 10 flies in a group could have max chasing events of 10 when they form a ring and the index is 100%. If only five chasing pairs are detected in a 10-fly group, the index is 50%.

All the pipeline codes and relevant algorithms for building the MAFDA platform were uploaded to the Zenodo platform at https://zenodo.org/record/7752914#.ZBkxBuzMLdo. Raw experiment data were uploaded to https://zenodo.org/record/7752914#.ZBkxBuzMLdo.

### Cuticular hydrocarbon analysis

Virgin male and female flies were collected at emergence and cultured at 29°C for 7 days. Each sample (including 10 flies) was frozen at −20°C for 15 min, and then introduced to a 2-ml vial containing 100 μl of hexane (Thermo Fisher Scientific, H303) with *n*-C19 alkanes (10 ng/μl; MilliporeSigma, N28906) as an internal standard. After extraction for 5 min, a 1-μl solution was injected into a GC (Trace 1310, Thermo Fisher Scientific) in splitless mode equipped with a TG-5MS capillary column (30 m by 0.25 mm by 0.25 μm; Thermo Fisher Scientific), using helium as carrier gas (1.0 ml/min). The column temperature was programmed to increase from 90° to 150°C at 20°C/min and then to 300°C at 3°C/min. The GC was coupled with a MS (ISQ 7000, Thermo Fisher Scientific). The injection temperature was 280°C, the MS source temperature was 310°C, and the transfer line was 300°C. The MS was set to scan a mass range from 40 to 550. A standard mixture of *n*-alkanes (C7 to C40, MilliporeSigma) was injected following the same temperature program. The identity of 7(*Z*)-tricosene, 11-*cis* vaccenyl acetate, and 7(*Z*),11(*Z*)-heptacosadiene was confirmed by comparison of retention times and mass spectra with synthetic standards (Cayman Chemical, catalog nos. 9000313, 10010101, and 10012567, respectively). Other compounds were tentatively identified on the basis of electron ionization mass spectra and Kovats indices, as well as previously published data in *Drosophila* (*103*, *104*). The quantities of CHCs were calculated on the basis of peak areas in comparison with the internal standard. For each analysis, five biological replicates and two technical replicates were conducted.

### RNA-seq and data analysis

Adult oenocytes were carefully dissected in 1× phosphate-buffered saline (PBS) before RNA extraction. Total RNAs were extracted from 7-day-old adult oenocytes using the Zymo RNA preparation kit. Two replicates were collected for each genotype. NEBNext Poly(A) mRNA Magnetic Isolation Module and NEBNext Ultra II RNA Library Prep Kit for Illumina were used for library preparation. The libraries were sequenced using an Illumina HiSeq 2500 system, obtaining 40 million reads for each sample.

Following the sequencing of samples, we used the fastp 0.22.0 tool with its default parameters to automatically trim the reads (*105*). This resulted in the retention of a vast majority of the bases and reads, with minimal differences among all libraries. Subsequently, we used bowtie2—an intron-aware short-read aligner—to map all reads to the dme6 reference genome (*106*). To quantify gene expression levels, we used RNA-Seq by Expectation-Maximization (RSEM) 1.2.31 and performed pairwise group comparisons using edger 3.36.0 on the output generated by RSEM (*107*). Significantly differentially expressed genes (DEGs) were selected on the basis of the threshold of *P* ≤ 0.001 and ≥4-fold change. The alignment of reads and the generation of the DEG matrix were carried out using the scripts provided by trinityrnaseq (*108*).

After generating the expression matrix, we performed sample clustering using PCA from the R package (*109*) and used the “pheatmap” function to generate heatmaps. We then used further downstream analyses, such as KEGG and GSEA (*63*), using clusterProfiler 4.2 (*110*). The KEGG pathway maps were visualized using pathview (*111*).

The RNA-seq data reported here have been deposited in National Center for Biotechnology Information’s Gene Expression Omnibus (GEO) and are accessible through GEO series accession number GSE227895.

### cDNA synthesis and RT-qPCR

Adult oenocytes were dissected in 1× PBS and stored at −80°C until all time points were collected. The tissue was homogenized with TRIzol (Thermo Fisher Scientific) using standard protocols on ice. Following phase separation, the aqueous phase was transferred into a new tube, mixed with an equal volume of 70% ethanol, and loaded directly onto the mini kit columns (Zymo RNA preparation kit). The remaining steps of the RNA isolation were performed in accordance with the manufacturer’s protocol, including on-column deoxyribonuclease digestion (Qiagen) for 15 min at room temperature.

Reverse transcription was performed on 0.25 to 1 μg of total RNA using the SuperScript Reverse Transcriptase II from Thermo Fisher Scientific (catalog no. 18064022) and oligo(dT) primers. cDNA was used as a template for qPCR.

RT-qPCR was performed with a QuantStudio 3 Real-Time PCR System and PowerUp SYBR Green Master Mix (Thermo Fisher Scientific). Three independent biological replicates were performed with three technical replicates each. The mRNA abundance of each candidate gene was normalized to the expression of *Rp49* by the comparative cycle threshold methods. Primer sequences are listed in table S5.

### Generation of anti-Fru^COM^ antibody (anti-Fru^ALL^)

The rabbit polyclonal antibody against Fru^COM^ was generated by ABclonal (Wuhan, China). Briefly, the fragment of the *fru* gene encoding 89 amino acids from the common part of the polypeptide, RERERERERERDRDRELSTTPVEQLSSSKRRRKNSSSNCDNSLSSSHQDR HYPQDSQANFKSSPVPKTGGSTSESEDAGGRHDSPLSMT, was cloned into the expression vector pET-28a (Sigma-Aldrich, #69864). A small ubiquitin-like modifier (SUMO) tagged Fru^COM^ fusion antigen was synthesized from bacteria, purified, and used to immunize the rabbit. The anti-Fru^COM^ antibody was affinity-purified. This polyclonal antibody recognizes an epitope in exon C3 of *fru*, which is present in both Fru^COM^ and Fru^M^ isoforms.

### Immunostaining and confocal imaging

Tissue samples were dissected in PBS, and then fixed in 4% formaldehyde in PBS for 20 min. After washing with PBS with 0.2% Triton X-100 (PBST), the samples were incubated in PBT with primary antibodies at 4°C overnight with shaking, and then washed in PBT three times for 15 min each. The following antibodies were used in immunostaining: anti-GFP (1:200; Cell Signaling Technology, #2956S), anti-hemagglutinin (HA) (1:500; Cell Signaling Technology, #3724S), anti-HNF4 guinea pig polyclonal antibody (1:100; a gift from G. Storelli) (*65*), anti-Fru^COM^ rabbit polyclonal antibody (1:500), and rabbit anti-Fru^M^ polyclonal antibody (1:250) (*59*). The secondary antibodies conjugated with Alexa Fluor 546 or 633 (Invitrogen) were diluted 1:200 and incubated at room temperature for 2 hours. Nuclei were labeled with 4′,6-diamidino-2-phenylindole (DAPI) (1:1000; Invitrogen, #D1306). After washing, samples were mounted and imaged with Zeiss LSM 800 or Zeiss LSM 980 Confocal Microscopes. Image analysis was performed in ImageJ.

### TAG assays

For TAG assays, 8 whole adult or 20 adipose tissues were homogenized in 100 μl of PBS, 0.5% Tween 20, and immediately incubated at 70°C for 5 min. Heat-treated homogenate (20 μl) was incubated with either 20 μl of PBS or Triglyceride Reagent (Sigma-Aldrich, T2449-10ML) for 30 min at 37°C, after which the samples were centrifuged at maximum speed for 3 min. Then, 30 μl was transferred to a 96-well plate and incubated with 100 μl of Free Glycerol Reagent (Sigma-Aldrich, F6428-40ML) for 5 min at 37°C. Samples were assayed using a BioTek Synergy HT microplate spectrophotometer at 540 nm. TAG amounts were determined by subtracting the amount of free glycerol in the PBS-treated sample from the total glycerol present in the sample treated with Triglyceride Reagent. TAG levels were normalized to protein amounts in each homogenate using a Bradford assay (Bio-Rad), and data were analyzed using a Student’s *t* test. Independent experiments were performed two to three times.

### Thin layer chromatography

The adipose tissue of 20 adult flies was homogenized in a 2:1:0.8 ratio of methanol:chloroform:water using 1.4-mm ceramic beads by vigorous vortexing for 10 min at 4°C. The samples were incubated in a water bath at 37°C for 1 hour. Next, chloroform and 1 M KCl were added to the sample in a 1:1 ratio, centrifuged at 3000 rpm for 2 min, and the bottom layer containing lipids was aspirated using a syringe. Lipids were dried using argon gas and resuspended in chloroform (100 μl of chloroform/7 mg of fly weight). Extracted lipids alongside serially diluted standard neutral lipids of known concentrations were separated on TLC plates using hexane:diethyl ether:acetic acid solvent (80:20:1). TLC plate was air-dried for 10 min, spray-stained with 3% copper(II) acetate in 8% phosphoric acid, and incubated at 180°C in the oven for 10 min to allow bands to develop for scanning and imaging.

### Starvation

Water was added to polystyrene vials (Genesee Scientific, catalog no. 32-110) until they were half full. A dense weave cellulose acetate stopper (Genesee Scientific, catalog no. 49-101) was then inserted into the bottom of the vial, allowing the artificial substrate to become saturated with water. Any excess water was discarded and newly emerged flies were transferred to these vials at a density of 5 to 10 males per vial. To reduce water evaporation, a second dense weave cellulose acetate stopper was used to seal the vials. Starvation experiments were conducted at 29°C. In this case, animals were transferred daily to fresh starvation vials and collected at 6 to 7 days after emergence for lipid stains.

### Lipid stains

Oenocytes dissected from adult flies were fixed in 4% paraformaldehyde for 30 min at room temperature, rinsed in PBS, and incubated in Bodipy 493/503 (1:1000; Thermo Fisher Scientific, catalog no. D3922) or Nile red (1:2000; TCI America, 7385-67-3) for 1 hour at room temperature, in the dark, to stain the neutral LDs. Next, the oenocytes tissues were rinsed in PBS and mounted in antifade reagent with DAPI (1:1000; Invitrogen, #D1306).

### Quantification and statistical analysis

Data analyses were conducted in GraphPad Prism and the R package. Unpaired *t* test was used for two-sample comparisons; one-way analysis of variance (ANOVA) followed by Dunnett's multiple comparison test and one-way ANOVA with Tukey’s multiple comparison test were used for multiple-sample comparisons. Specific statistical approaches for each figure are indicated in the figure legend. Nonnormally distributed data, the foraging index, for instance, were analyzed using the Wilcoxon rank sum test to determine the significant difference between groups. The statistical test used for each figure is clarified in the figure legend. The raw mean, SEM, and *P* value are presented in supplementary tables.

## Notes

View/request a protocol for this paper from *Bio-protocol*.
