# Supplementary material for: Integrating lipid metabolism, pheromone production and perception by Fruitless and Hepatocyte Nuclear Factor 4
Source: Sci Adv. 2023 Jun 30;9(26):eadf6254. doi: 10.1126/sciadv.adf6254 (PMC10313179; doi:10.1126/sciadv.adf6254)
Supplement: Supplementary file 1 — Figs. S1 to S9 Legends for tables S1 to S7 Legends for movies S1 to S5 [file sciadv.adf6254_sm.pdf]

Science Advances
AAAS

Supplementary Materials for

Integrating lipid metabolism, pheromone production and perception by Fruitless and Hepatocyte Nuclear Factor 4

Jie Sun *et al.*

Corresponding author: Wu-Min Deng, wdeng7@tulane.edu

Sci. Adv. **9**, eadf6254 (2023)
DOI: 10.1126/sciadv.adf6254

The PDF file includes:

Figs. S1 to S9
Legends for tables S1 to S7
Legends for movies S1 to S5

Other Supplementary Material for this manuscript includes the following:

Tables S1 to S7
Movies S1 to S5

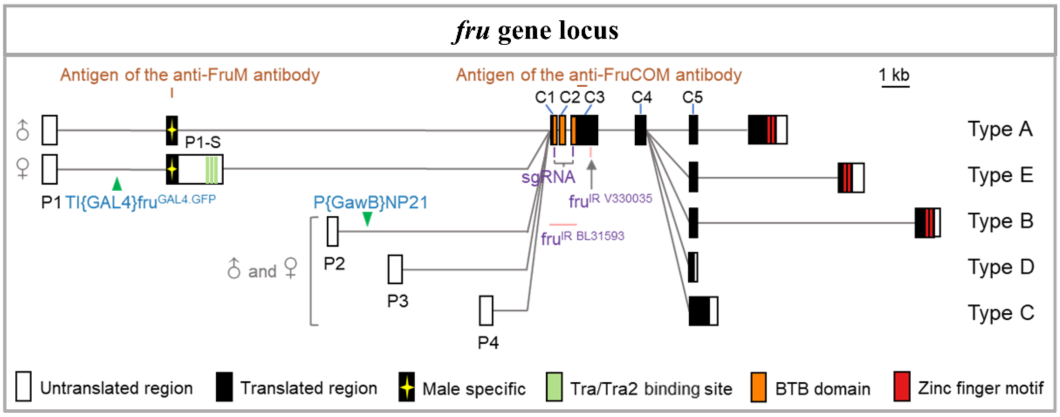

Fig. S1. The *fru* gene locus. Schematic representation of the *fru* gene locus. Locations of four promoters (P1–P4), the exon-intron organization, and the P-element insertion sites of fru^{P1} and fru^{NP21} (green triangles) are shown. Filled and open boxes indicate coding and non-coding exons, respectively. A-E denote isoform-specific exons for types A-E. The start and termination codons are also shown. The regions containing epitopes for the anti-Fru antibodies are indicated. *fru* gRNA and *fru* dsRNA sites are displayed.

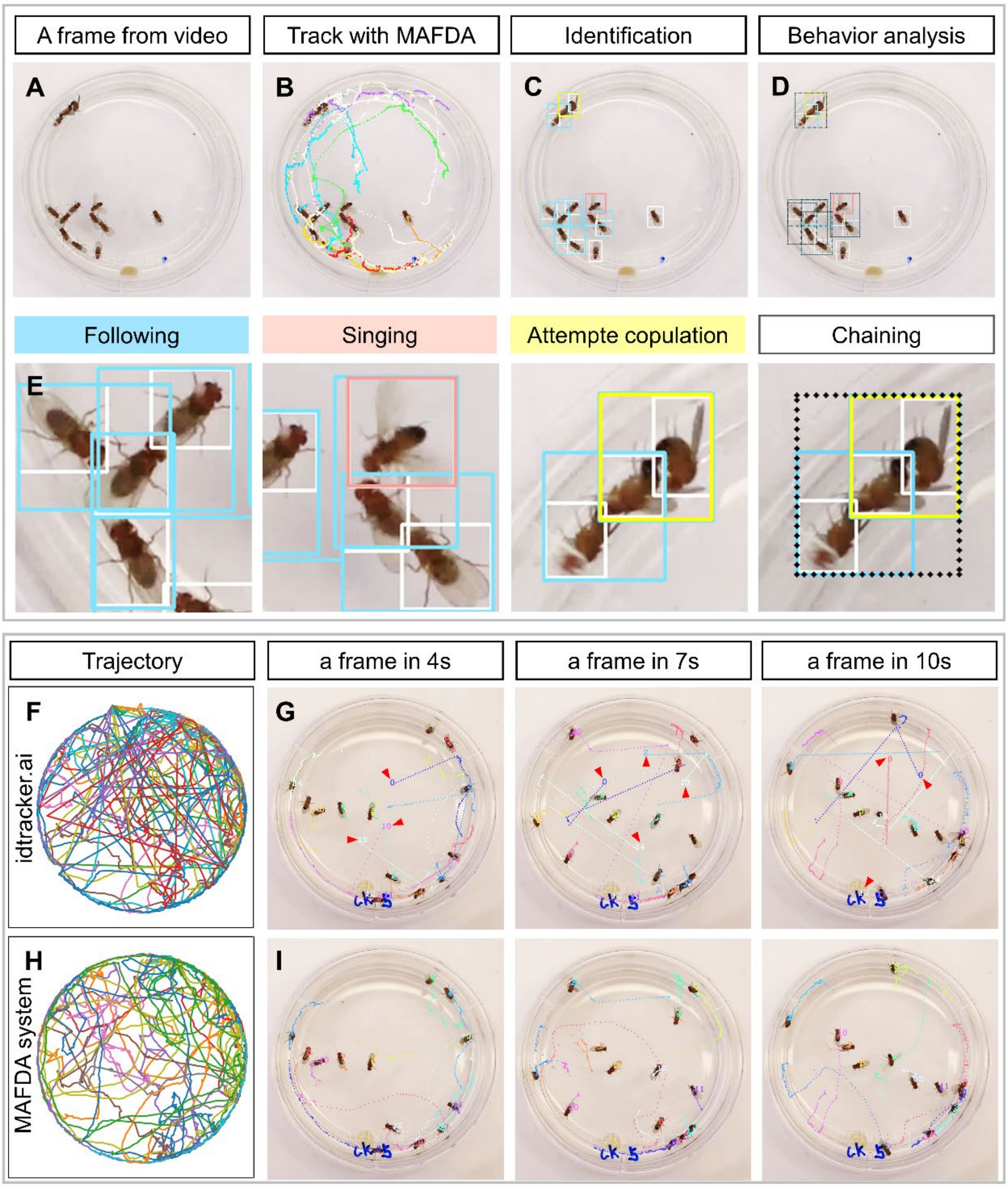

Fig. S2. The machine-learning-based automatic fly-behavioral detection and annotation (MAFDA) system. (A) A snapshot of the original video. (B) The tracked trajectory of individual flies indicated by different colored lines. (C-D) Identification of different behavior types for auto-annotation. (E) Different colors represent different behavior types. White boxes mark flies in resting/walking. Blue boxes mark flies that are following. Salmon box shows singing flies. Yellow box shows flies in copulation. Black dashed boxes mark chaining flies. (F) Fly trajectories identified by idtracker.ai. (G) Representative real-time trajectory video screenshots via idtracker.ai. The red arrow represents object loss. (H) Behavioral trajectories tracked using MAFDA. (I) Representative real-time trajectory video screenshots identified by MAFDA.

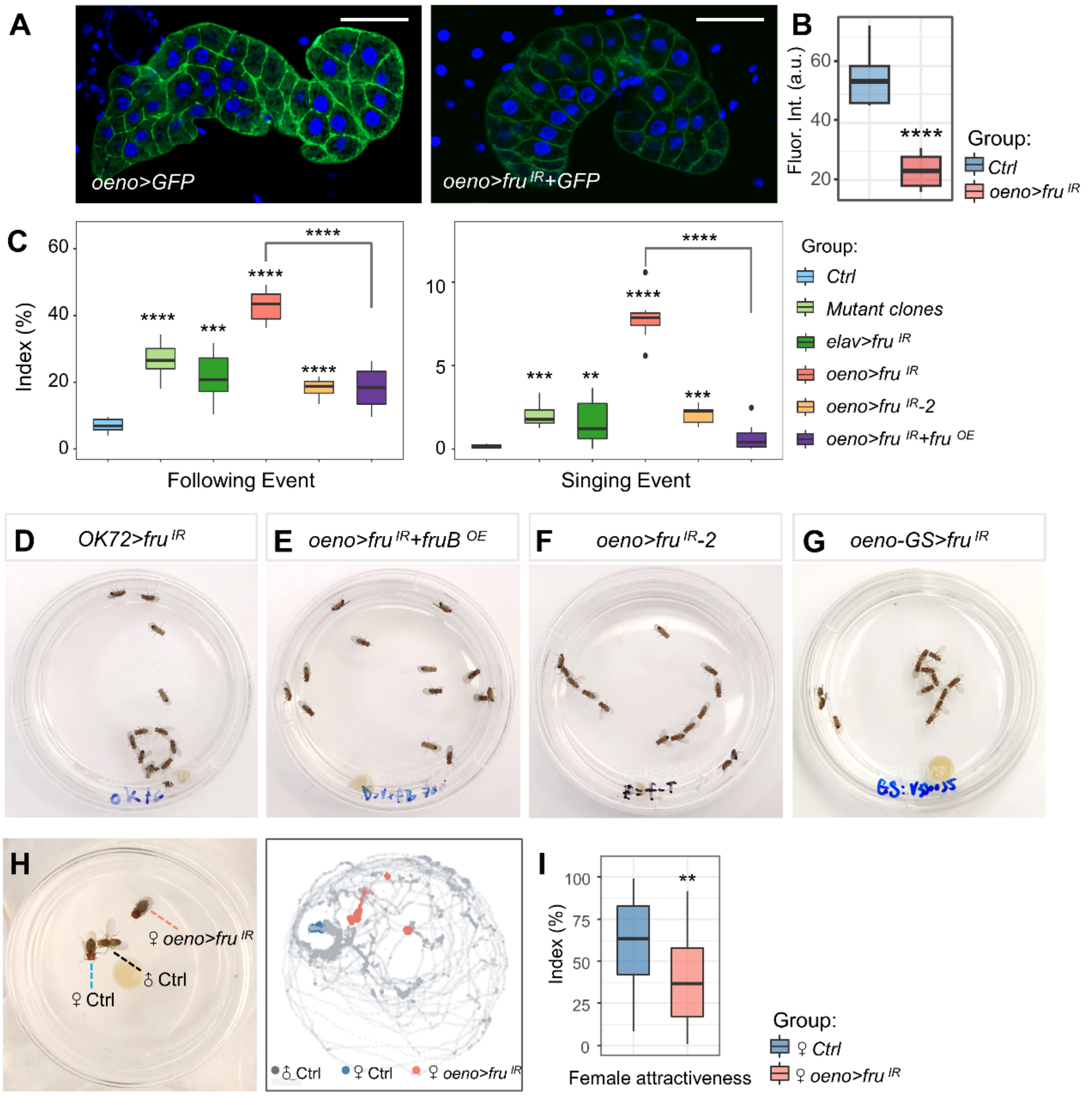

Fig. S3. Fly behavioral phenotyping of *fru* knockdowns in oenocytes. (A) Comparison of GFP signals in oenocytes using the *promE-Gal4 >UAS-GFP* with (right panel) or without (left panel) *fru*-knockdown (fru^{IR}). Scale bar, 10 μm. (B) Fluorescence intensity analysis of the control (*oeno>GFP*) and *fru*-knockdown ($oeno{>}fru^{IR}{+}GFP$) oenocytes. (C) Quantitative statistical results of following and singing index for Fig.1A. There were 13 flies per video with 8 independent biological replicates. (D-G) Representative video screenshots of each fly group with indicated genotype. (D) Using another oenocyte-Gal4 driver (*OK72-Gal4*) to knockdown of *fru* in males also resulted in male-male courtship. (E) Fru^{COMB} overexpression alleviates the defective behavior caused by *fru* knockdown in oenocyte. (F) Male flies with knockdown of *fru* using an alternative *RNAi* (BDSC#31593) in oenocytes also exhibits the male-male chaining behavior. (G) Using an oenocyte-specific GeneSwitch driver (*promE-GS-Gal4*), knockdown of *fru* in adult oenocytes induced male-male chaining behavior. (H) Oenocyte-specific *fru*-depletion in female flies reduced their sexual attractiveness to males. An event map generated from a 1-hour video of the two-choice courtship assay. Grey dots

indicate the *control* (*oeno*/+) male, blue dots indicate the headless *control* (*oeno*/+) female. Salmon dots indicate the headless *oeno>fru*^{IR} female. (I) Boxplots show that *oeno>fru*^{IR} females have lower courtship index (CI) than its heterozygote parental control lines (n=36), as revealed in the two-choice courtship assay. All data are represented as mean ± SEM. P values are calculated using one-way ANOVA followed by Holm-Sidak multiple comparisons. n.s., not significant, *p<0.05, **p<0.01, ***p<0.001.

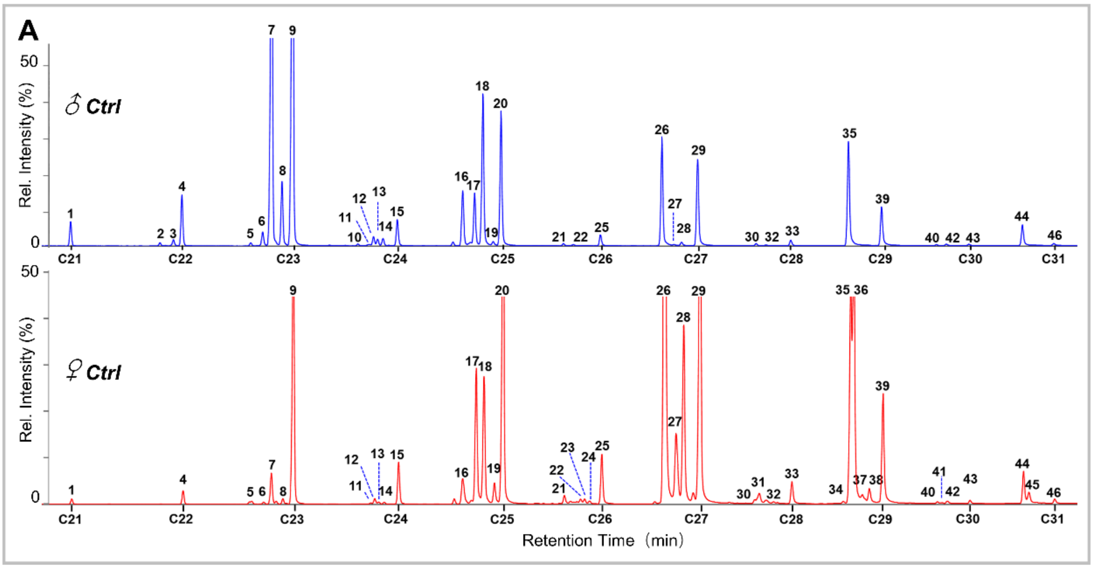

B

No.	Retention Time (min)	Compound	Chemical Class	Kovats index	No.	Retention Time (min)	Compound	Chemical Class	Kovats index
1	22	n-Heneicosane	alkane	2100	23	34.628	(Z)-9-Hexacosene	monoene	2582
2	24.203	(+)-6-Docosene	monoene	2180	24	34.753	(Z)-7-Hexacosene	monoene	2588
3	24.530	cis-vaccenyl acetate	cVA	2192	25	35.053	n-Hexacosane	alkane	2600
4	24.741	n-Docosane	alkane	2200	26	36.553	7,11-C27:2 (7,11-HD) / 2-Me-C27	diene / Me-alkane	2663
5	26.438	7,11-C23:2 (7,11-TD)	diene	2262	27	36.849	(Z)-9-Heptacosene	monoene	2675
6	26.727	(Z)-9-Tricosene	monoene	2273	28	37.036	(Z)-7-Heptacosene	monoene	2683
7	26.962	(+)-7-Tricosene	monoene	2281	29	37.437	n-Heptacosane	alkane	2700
8	27.206	(+)-5-Tricosene	monoene	2290	30	38.818	7,11-C28:2 (7,11-OD)	diene	2760
9	27.475	n-Tricosane	alkane	2300	31	38.927	2-Me-C27	Me-alkane	2765
10	29.074	x,y-C24:2	diene	2362	32	39.090	Octacosene	monoene	2774
11	29.332	3-Me-C23	Me-alkane	2372	33	39.733	n-Octacosane	alkane	2800
12	29.455	(+)-7-Tetracosene	monoene	2377	34	40.998	9,13-C29:2	diene	2856
13	29.557	(+)-6-Tetracosene	monoene	2381	35	41.175	2-Me-C28	Me-alkane	2864
14	29.689	(Z)-5-Tetracosene	monoene	2386	36	41.256	7,11-C29:2 (7,11-ND)	diene	2868
15	30.043	n-Tetracosane	alkane	2400	37	41.467	(Z)-9-Nonacosene	monoene	2877
16	31.652	7,11-C25:2 (7,11-PD)	diene	2463	38	41.644	(Z)-7-Nonacosene	monoene	2885
17	31.948	(x)-9-Pentacosene	monoene	2474	39	41.981	n-Nonacosane	alkane	2900
18	32.158	(Z)-7-Pentacosene	monoene	2483	41	43.416	2-Me-C29	Me-alkane	2967
19	32.403	(x)-5-Pentacosene	monoene	2492	43	44.113	n-triacontane	alkane	3000
20	32.601	n-Pentacosane	alkane	2500	44	45.426	2-Me-C30	Me-alkane	3063
21	34.131	2-Me-C25	Me-alkane	2563	45	45.566	7,11-C31:2	diene	3070
22	34.532	7,11-C26:2	diene	2574	46	46.198	n-Hentriacontane	alkane	3100

Fig. S4. GC-MS analysis of cuticular hydrocarbon extracts from control males and females. (A) Sexually dimorphic CHC profiles in *Drosophila melanogaster*. The graphs show representative chromatograms of CHCs of 7-day-old virgin male and female flies, with the male at the top (blue) and female at the bottom (red). Compounds corresponding to each numbered peak are listed in B. Compounds that are shared between sexes bear the same number. The identity of each hydrocarbon was confirmed by comparison with synthetic standards and Kovats index in publication *(103, 104)*.

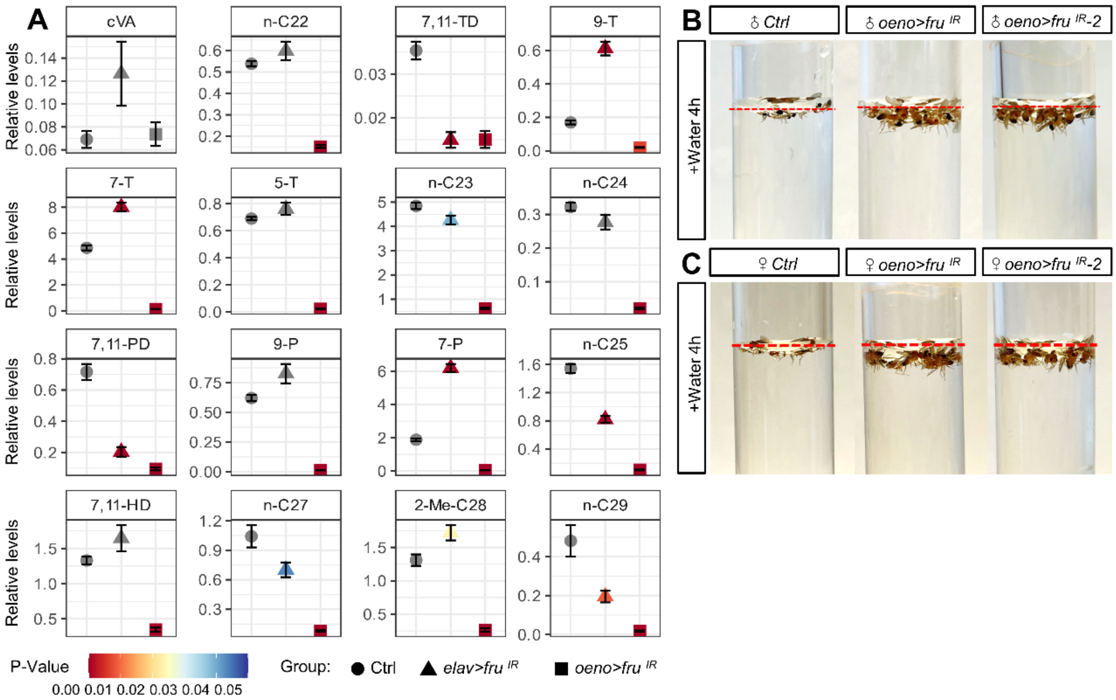

Fig. S5. Oenocyte-specific knockdown of *fru* decreased representative cuticular hydrocarbons (CHCs) and waterproof capacity. (A) Sixteen representative CHCs were selected for quantitative analysis and normalized by the internal standards. Five independent biological replicates were collected for each genotype. Circles indicate the *control* group, triangles indicate the *elav>fru^{IR}* group and squares indicate the *oeno>fru^{IR}* group. Different p-values are shown by gradient colors. (B and C) The cuticular hydrophobicity of control, *oeno>fru^{IR}* and *oeno>fru^{IR}-2* flies were tested following death by dry starvation and incubation of the carcasses in water with agitation. After 4 hours, dead control carcasses continued to float above the liquid (represented by the red dotted line), while *oeno>fru^{IR}* and *oeno>fru^{IR}-2* equilibrated below the surface (n=13 males in each vial). Males are shown in B and females in C.

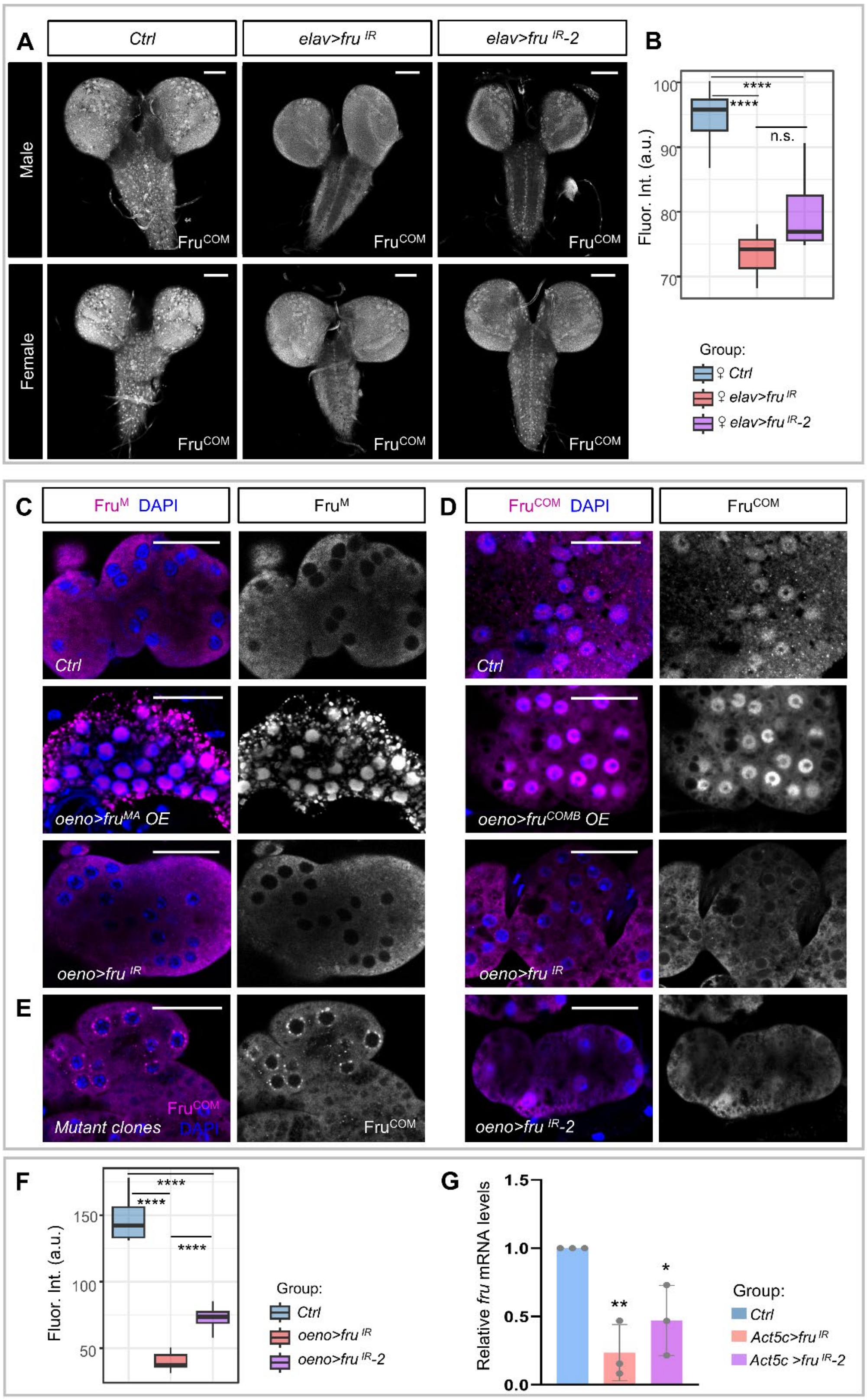

Fig. S6. Fru expression in oenocytes and validation of the anti-Fru^{COM} antibody. (A) Validation of the anti-Fru^{COM} antibody by staining the central nervous system of the 3rd-instar larvae of both sexes. Scale bar, 100 µm. (B) Fluorescence intensity analysis to show reduced anti-Fru^{COM} signals when *fru* was knocked down with *elav>Gal4*. Two independent RNAi

lines showed different knockdown efficiency. (C and D) Anti-Fru antibodies were used to stain oenocytes from the control wild-type, overexpression of Fru^{M}/Fru^{COM} or knockdown of Fru (all isoforms) in oenocytes. Scale bar, 10 µm. (E) anti-Fru^{COM} signal was lost in the oenocyte nuclei of *fru* MAGIC clones. Scale bar, 10 µm. (F) Quantitative analysis of Fru^{COM} fluorescence intensity in *oeno>fru*^{IR}. (G) Transcript levels of *fru* were analyzed by RT-qPCR in whole-body driven *fru RNAi* from different sources. Transcript levels were normalized to Rp49 mRNA and presented relative to control levels. Data are represented as mean ± SEM. P values are calculated using one-way ANOVA followed by Holm-Sidak multiple comparisons. Asterisks indicate statistically significant differences between conditions. n.s., not significant, *p<0.05, **p<0.01, ***p<0.001

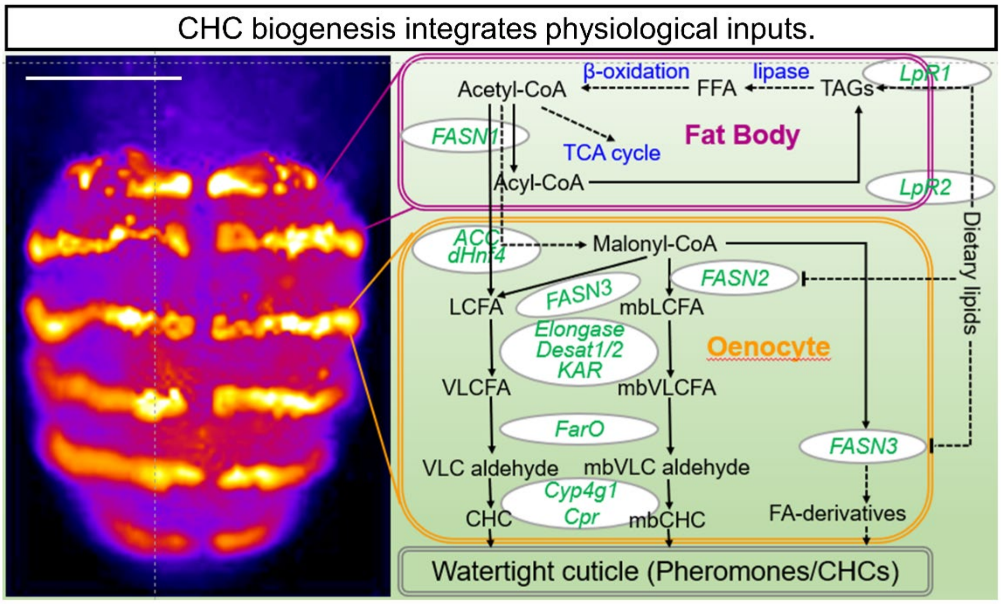

Fig. S7. A schematic diagram of the cuticle hydrocarbon (CHC) biogenesis pathway. The biosynthesis of CHC is associated with the fatty acid metabolism. Free fatty acids are converted into hydrocarbons by a series of enzymatic reactions. A key enzyme in CHC biosynthesis in *Drosophila* is fatty acid synthase (FASs), which catalyzes the production of fatty acids. The conversion of fatty acids into a variety of hydrocarbons involves enzymes such as acetyl-CoA carboxylase (ACC), multiple elongases and desaturases (Desat1/2). Acetyl-CoA carboxylase converts acetyl-CoA into malonyl-CoA, which is used as a precursor for the synthesis of various types of hydrocarbons. Desaturases introduce double bonds into hydrocarbon chains, resulting in the production of different types of unsaturated hydrocarbons. Subsequently, the terminal oxidative decarbonylation of CHC is carried out by a functionally conserved P450 enzyme (Cyp4g1). Scale bar, 500 µm.

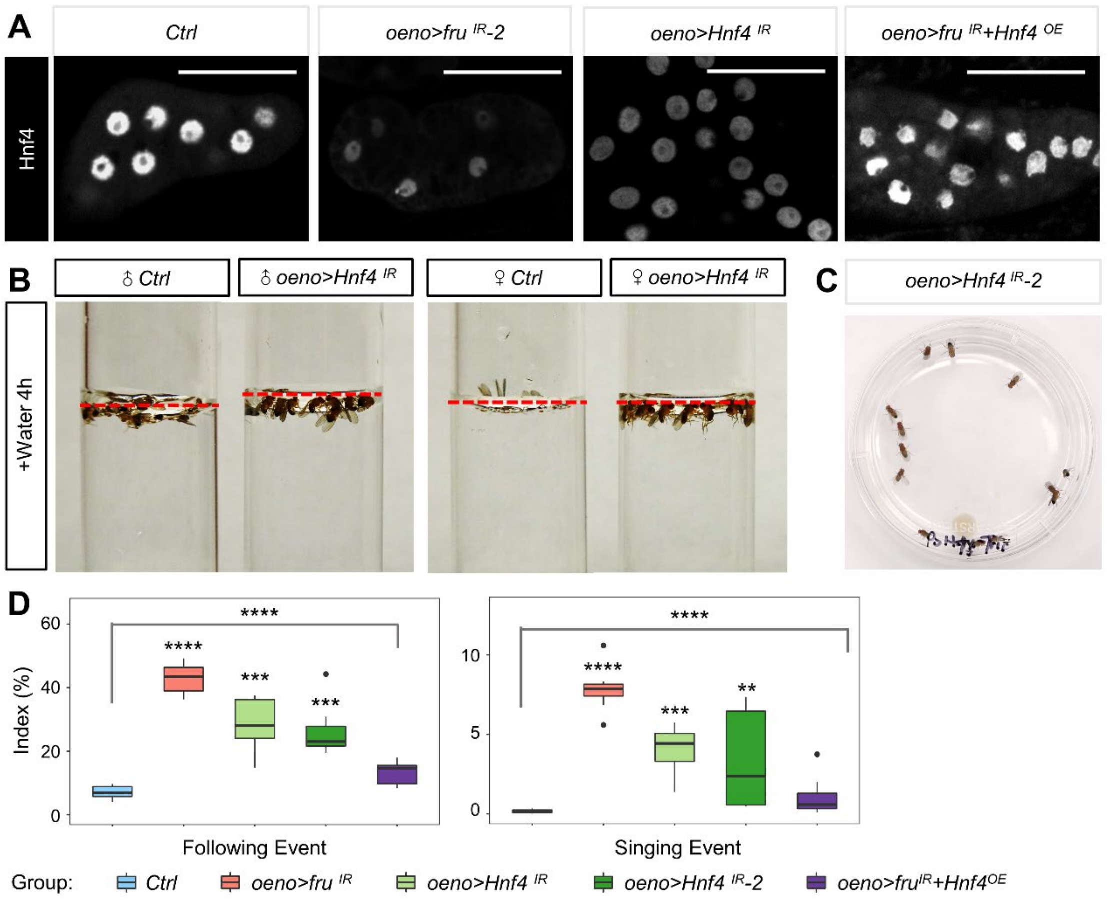

Fig. S8. *Hnf4* knockdown in oenocytes resulted in behavioral and CHC synthesis defects. (A) Using anti-HNF4 to detect the protein level of HNF4 in *oeno>fru*^{IR}*-2, oeno>Hnf4*^{IR} and *oeno>fru*^{IR}*+Hnf4*^{OE}. Scale bar, 10 µm. (B) The cuticular hydrophobicity of control and *oeno>Hnf4*^{IR} was tested following death by dry starvation and incubation of the carcasses in water with agitation. After 4 hours, dead control carcasses continued to float above the liquid (represented by the red dotted line), while *oeno>Hnf4*^{IR} equilibrated below the surface in both sexes (n=13 files in each vial). (C) A representative video screenshot of group-housed *oeno>Hnf4*^{IR}*-2* flies. Knockdown of *Hnf4* in oenocytes with a different *Hnf4 RNAi* (BDSC#64988) also showed the male-male chaining behavior. (D) Quantitative analysis of the following and singing behaviors displayed by flies with different genotypes from Fig. 6C. Thirteen flies were used per video with eight independent biological replicates. Data were represented as mean ± SEM. P-values were calculated using one-way ANOVA followed by Holm-Sidak multiple comparisons. ***p<0.001.

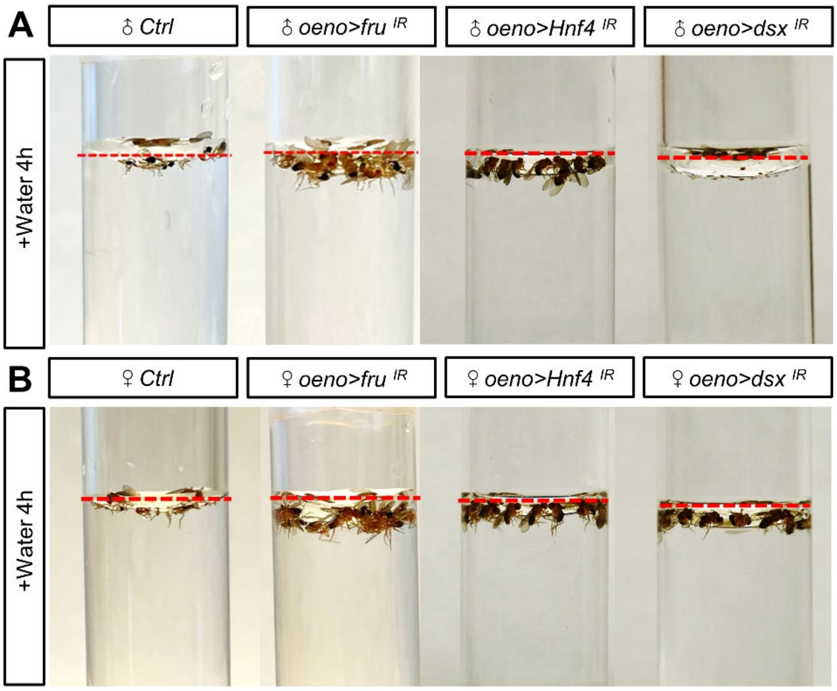

Fig. S9. Depletion of *dsx* in oenocytes resulted in sexually dimorphic water resistance. (A and B) The cuticular hydrophobicity of *oeno>dsx*^{IR} was tested following death by dry starvation and incubation of the carcasses in water with agitation. After 4 hours, dead *oeno>dsx*^{IR} male carcasses continued to float above the liquid (represented by the red dotted line), while *oeno>dsx*^{IR} females equilibrated below the surface (n=13 males in each vial).

Other supplementary material for this manuscript includes the following:

Table. S1 Behavioral analysis statistics.

Table. S2 Fluorescence intensity index analysis.

Table. S3 CHC profiles and raw reads of GC-MS analysis.

Table. S4 Statistics of RNA-seq read mapping.

Table. S5 The list of PCR primers and RT-qPCR analysis.

Table. S6 TAG level statistics.

Table. S7 Key resources table.

Movie. S1 The video of *fru* MAGIC mutants.

Movie. S2 The video of male behavior in nervous system knockdown of *fru*.

Movie. S3 The video of male behavior in oenocytes knockdown of *fru*.

Movie. S4 The video of *Hnf4* knockdown in oenocytes.

Movie. S5. The video of $oeno>fru^{IR+}Hnf4^{OE}$ males.
